# Hypoxia response in glioblastoma cells: effect of trehalose on macropinocytosis, autophagy and cell survival

**DOI:** 10.1016/j.bbrep.2025.102284

**Published:** 2025-10-02

**Authors:** Barbara Del Bello, Cristina Ulivieri, Emilia Maellaro

**Affiliations:** a Department of Molecular and Developmental Medicine, University of Siena, via A. Moro, 53100, Siena, Italy; b Department of Life Sciences, University of Siena, via A. Moro, 53100, Siena, Italy

**Keywords:** Hypoxia, Glioblastoma cells, Macropinocytosis, Autophagy, Trehalose

## Abstract

In glioblastoma multiforme, the most malignant brain tumor in adults, the hypoxic milieu is believed to contribute to tumor aggressiveness and resistance to therapy. Here, human glioblastoma U373-MG and T98G cells were exposed to hypoxia (1 % oxygen) or normoxia (18 % oxygen), and treated with trehalose, a natural disaccharide increasingly studied for its therapeutic potential in cancer. In all samples under hypoxia, HIF-1α stabilization was accompanied by a decrease in Nrf2 and p62/SQSTM1 proteins; redox imbalance also occurred, as documented by increased levels of ROS and parallel lowering of glutathione. Trehalose treatment increased Nrf2 and p62 proteins under normoxia, an effect lost or downsized under hypoxia. Differently, under both oxygen concentrations, trehalose increased glutathione content, consistently with its antioxidant role. In U373-MG cells, trehalose induced remarkable macropinocytosis under hypoxia, albeit less than under normoxia; on the contrary, in autophagy-proficient T98G cells, trehalose further increased the autophagic process under hypoxia compared to normoxia. As regards long-term cell fate (evaluated as colony-forming capacity), hypoxia only proved to be a favorable condition for T98G cells. However, in both cell lines, trehalose treatment significantly and dose-dependently decreased clonogenic capacity under normoxia and hypoxia; in particular, the long-lasting stimulation of macropinocytosis in U373-MG cells induced extensive cell death by methuosis. Overall, our findings suggest that trehalose-induced macropinocytosis or autophagy could also play a tumour-suppressive role in the hypoxic tumor milieu that characterizes glioblastoma, making its synergy with conventional chemotherapy and radiotherapy worth exploring.

## Highlights

• Hypoxia upregulates HIF-1α but downregulates Nrf2 and p62/SQSTM1 in human glioblastoma U373-MG and T98G cells.
• Trehalose enhances GSH levels and stimulates macropinocytosis or autophagy under normoxia and hypoxia.
• Trehalose significantly decreases the clonogenic capacity of both cell lines.
• Trehalose induces cell death by methuosis in U373-MG cells under normoxia and hypoxia.

## 1 Introduction

Glioblastoma multiforme (GBM) (grade IV astrocytoma, according to World Health Organization classification) is the most common brain tumor in adults. It is highly aggressive, chemo- and radio-resistant, and relapse-prone. To date, the standard therapeutic approach, i.e. surgical removal followed by radiotherapy plus alkylating chemotherapy with temozolomide, elicits a very poor response, with a median survival of less than two years [1]. Much effort has been made by researchers to find effective new therapeutic agents, to use alone or in combination with standard treatments. It is a feature of basic oncology research that the greater part of experimental data is obtained in cell line cultures or in cultured cells isolated from biopsies, with the limitation that to be effective against glioblastoma *in vivo,* the agents must be able to cross the blood-brain barrier (BBB). A further important difference between *in vitro* conditions and *in vivo* tumor cells is the oxygen level. In the overwhelming majority of *in vitro* studies, cells are grown under standard air conditions, i.e. humidified atmosphere with 5 % CO_2_. This condition, corresponding to an oxygen concentration of approximately 18 %, is regarded as “normoxia”. However, it is far from normal in living tissues, where a mean value of around 5 % is estimated in most mammalian tissues. An oxygen concentration of 18 % is therefore a hyperoxic condition which could potentially affect a variety of biological processes. Accordingly, the term “physioxia” was recently introduced for such naturally occurring “physiological hypoxia” [2]. This new perspective is even more relevant for solid tumors, most of which are chronically exposed to even lower oxygen levels (ranging from 0.5 % to 2 %) than the corresponding normal tissue [[3], [4], [5]].

Macroautophagy (commonly referred to as autophagy) and macropinocytosis are two complex cell processes that share the capacity of providing cells with nutrients. They prove to be particularly important in the context of tumor cells. Autophagy begins with the biogenesis of double-membrane vesicles (autophagosomes) derived from the endoplasmic reticulum and other organelles, which engulf intracellular cargo, including misfolded protein aggregates, dysfunctional organelles and ribosomes. After fusion of the loaded autophagosomes with lysosomes, the cargo is broken down and the products recycled [6]. Macropinocytosis is an actin-dependent and clathrin-independent endocytic pathway, through which cells non-selectively internalize large portions of extracellular fluid in single-membrane endocytic vesicles termed macropinosomes. Trafficking in the cytosol, macropinosomes can fuse with lysosomes to break down their cargo [7]. By supplying basic components for *de novo* building of macromolecules and for metabolism, autophagy and macropinocytosis both allow cells to survive in nutrient-poor conditions. As regards macropinocytosis, added value is derived from the ingestion of extracellular material, which increases net cellular biomass and provides supplementary nutrients to fuel high metabolic demand. In this perspective, intrinsic cellular capacity to perform autophagy or macropinocytosis can be expected to play a tumor-promoting role. On the other hand, these processes attracted increasing pharmacological interest for tumor cell killing. In fact, when autophagy or macropinocytosis become excessive and/or long-lasting, they prove to be harmful to cell viability by inducing autophagy-dependent or -mediated cell death [8], or cell death by methuosis [9].

Trehalose is a natural non-reducing disaccharide consisting of two d-glucose residues linked by an α,α–1,1 glycosidic bond. Many organisms, including plants, bacteria, fungi, insects, and other invertebrates, synthesize trehalose. For humans, who do not synthesize it, the major natural dietary sources of trehalose are mushrooms, honey, shrimps, and other foods produced with the use of baker's or brewer's yeast [10]. Trehalose manufactured by enzymatic technology was listed *Generally Recognized As Safe* (GRAS) by the U.S. Food and Drug Administration in 2000; in 2001, the EU Scientific Committee on Food also declared trehalose safe, and in the following years many other countries also authorized the marketing of trehalose as a novel supplement in food products, medications, and cosmetics. Over the last 15 years, trehalose has attracted attention for its therapeutic potential in several diseases, including cancer [11,12], and due to its capacity to cross the blood-brain barrier (BBB) [13] in the case of neurodegenerative diseases [14,15].

We recently showed that treatment with trehalose in standard culture conditions (18 % oxygen) induces a sustained autophagic response in human glioblastoma T98G cells and extensive macropinocytosis in human glioblastoma U373-MG cells [16]. The long-term effect of trehalose on both cell lines (evaluated as colony-forming capacity) is remarkable; in particular, in NF1-deficient U373-MG cells showing RAS hyperactivity, sustained macropinocytosis eventually leads to cell death by methuosis.

Against this background, we considered trehalose worthy of further study in glioblastoma cells under naturally occurring oxygen conditions. Since the average oxygen level is estimated to be below 2 % in glial tumors [4,5], we performed experiments on human glioblastoma U373-MG and T98G cells treated with trehalose under 1 % oxygen compared to 18 % oxygen. In both conditions, we evaluated some parameters commonly considered hypoxia-related, including HIF and Nrf2 activation and cell redox state. We also evaluated how hypoxia impacts trehalose-induced autophagy and macropinocytosis, and how these cell events in turn impact the long-term fate of cells. Since trehalose treatment significantly reduces clonogenic capacity and induces cell death by methuosis in U373-MG cells under the physiological low-oxygen conditions characteristic of glioblastoma, its potential synergy with standard chemotherapy and radiotherapy warrants further investigation in both cell culture and *in vivo* studies.

In all subsequent sections we have used canonical terms for oxygen concentration, i.e. normoxia for standard cell cultures with 18 % oxygen, and hypoxia for cell cultures with 1 % oxygen.

## 2 Materials and methods

### 2.1 Cell cultures and treatments

The human glioblastoma cell lines, U373-MG and T98G, originally provided by ECACC, were kindly gifted by Prof. S. Comincini (University of Pavia, Italy). Cell lines were confirmed negative for Mycoplasma contamination by periodic checks with the MycoAlert Mycoplasma detection kit (LTO7-218, Lonza Rockland). Both cell lines were routinely grown in RPMI 1640 medium (Merck), containing 10 % heat-inactivated fetal bovine serum (FBS) (Euroclone), 2 mM glutamine (Merck), and 50 mg/L gentamycin (Merck), at 37 °C, in a humidified atmosphere with 5 % CO_2_, in an *incubator HERAcell Heraeus* (Hanau, Germany). Cells were routinely harvested by a brief incubation in 0.05 % trypsin-0.02 % EDTA solution (Merck) and reseeded before reaching confluence. Harvested cells were manually counted in a Bürker chamber, and cell viability was assessed by the trypan blue exclusion assay.

For experiments, after an overnight resting of seeded cells, the medium was changed and cells were left untreated or treated with 90 mM (unless otherwise stated) trehalose (Merck), dissolved in complete medium. After treatment, cells were incubated for 48 h (unless otherwise stated) under two oxygen concentrations, in parallel: *i)* approximately 18 % atmospheric O_2_ (hereafter referred to as normoxia), in the above mentioned incubator routinely used for cell culturing; and *ii)* 1 % O_2_ (hereafter referred to as hypoxia), in an incubator ICO105 Memmert GmbH (Buchenbach, Germany), where O_2_ tension is maintained constant by automatic N_2_ injection in the chamber. At the end of experiments, hypoxic samples were very promptly processed to minimize cell culture reoxygenation.

### 2.2 Western blot analysis

For all proteins to be analysed except HIF-1α, cells were harvested by trypsinization, and resuspended in the following ice-cold lysis buffer: 20 mM HEPES, pH 7.5, containing 10 % glycerol, 0.1 % CHAPS, 0.2 % NP-40, 1 mM EDTA, 5 mM dithiothreitol (DTT), freshly prepared 1 mM phenylmethylsulphonyl fluoride (PMSF), protease inhibitor cocktail (Merck), and phosphatase inhibitors (1 mM Na_3_VO_4_, 10 mM NaF, and 10 mM β-glycerophosphate). After sonication on ice for 10 s (Vibracell, amplitude 60, 25 W) and centrifugation at 12,000*g* for 10 min at 4 °C, aliquots of the supernatants were assayed for protein concentration by using the Bradford reagent (B6916, Merck); other aliquots were mixed with Laemmli loading buffer 4X (Bio-Rad Laboratories) and the mixture warmed at 95 °C for 7 min. Equal amounts of protein (15–20 μg) were separated by sodium dodecyl sulphate polyacrylamide gel electrophoresis (SDS-PAGE) on 4–20 % Mini-Protean Precast gels (Bio-Rad Laboratories), for 60 min at 140 V, and electrophoretically transferred onto 0.22 μm nitrocellulose membranes (Bio-Rad Laboratories) for 90 min at 260 mA. Before adding primary antibodies, quality control and transfer efficiency were assessed by reversible membrane staining with Ponceau S (P7170, Merck) (0.1 % in 10 % acetic acid). After destaining with 0.01 M NaOH and rinsing with deionized water, nitrocellulose membranes were blocked for 1 h at RT in 10 % non-fat milk in phosphate-buffered saline (PBS)-0.05 % Tween-20 (PBST), and probed overnight at 4 °C with the following primary antibodies: anti–HIF–1α (1:1250, D1S7W, Cell Signaling Technology), anti-Lamin B1 (1:600, C-5, sc-365962, Santa Cruz Biotechnology), anti-GAPDH (1:2500, 0411, sc-47724, Santa Cruz Biotechnology), anti-Nrf2 (1:300, H-6, sc-518033, Santa Cruz Biotechnology), anti–HO–1 (1:1250, P249, Cell Signaling Technology), anti-LC3A/B (referred to as LC3) (1:1000, L7543, Merck), and anti-p62/SQSTM (1:500, D3, sc28359, Santa Cruz Biotechnology). All primary antibodies were diluted in 2 % BSA and 0.05 % sodium azide in PBST. After four washes with PBST, membranes were incubated for 1 h at RT with horseradish peroxidase-conjugated goat secondary antibodies anti-rabbit (R4880, Merck) or anti-mouse (A5420, Merck), diluted in 1 % non-fat milk in PBST. Proteins were visualized by chemiluminescence (Clarity Western ECL Substrate, Bio-Rad Laboratories) with a CCD camera gel documentation system (ChemiDoc™ XRS+, Bio-Rad Laboratories). Densitometric analysis of protein bands of interest was carried out using Image Lab software (Bio-Rad Laboratories); protein band intensity was normalized to the total amount of protein of each lane, as quantified using Image Lab software on Ponceau S-stained membrane.

As regards HIF-1α evaluation in hypoxic as well as normoxic samples, cells were promptly processed, and stringent precautions were also taken to avoid culture reoxygenation and oxygen-mediated HIF-1α degradation: cells were washed with *nitrogen-*purged ice-cold PBS and lysed directly in culture flasks with the following buffer: 20 mM TRIS-HCl pH 7.4, containing 137 mM NaCl, 2 mM EDTA, phosphatase inhibitors (0.5 mM Na_3_VO_4_, 1 mM NaF), 1 % NP-40, 10 % glycerol, and protease inhibitor cocktail. After 10 min on ice, cells were scraped, passed through a 19-gauge syringe needle, sonicated for 5 s, and centrifuged at 18,000*g* for 15 min at 4 °C. Aliquots of supernatant were mixed with Laemmli loading buffer 4X and the mixture warmed at 70 °C for 10 min.

### 2.3 Preparation of nuclear and post-nuclear fractions

Nuclear (N) and post-nuclear (pN) fractions were prepared essentially as previously described [17]. Briefly, cells were quickly washed in ice-cold PBS, then harvested by trypsinization and resuspended in the following hypotonic buffer: 10 mM HEPES, pH 7.9, 10 mM KCl, 1.5 mM MgCl_2_, containing 1 mM EGTA, 1 mM phenylmethylsulphonyl fluoride (PMSF), protease inhibitors cocktail, and phosphatase inhibitors (2 mM Na_3_VO_4_ and 1 mM NaF). After swelling for 15 min on ice, 10 % NP-40 was added (1/16 of the cell resuspension volume) and the homogenate was vigorously vortexed three times for 10 s. After checking under the microscope for the absence of intact cells, cell lysate was centrifuged at 12,000*g* for 60 s at 4 °C. The recovered supernatant was the post-nuclear fraction. The pellet was washed and resuspended in ice-cold lysis buffer, sonicated for 10 s, and centrifuged at 12,000*g* for 2 min; the obtained pellet was the nuclear fraction. After withdrawal of aliquots for protein assay, lysates were mixed with Laemmli loading buffer 4X and the mixture warmed at 95 °C for 7 min. Equal amounts of protein (20 μg) for nuclear fraction, post-nuclear fractions and total cell lysate were separated by SDS-PAGE.

### 2.4 Measurement of intracellular ROS formation in intact cells

Intracellular production of ROS was measured by DCF assay. The cell-permeable 2′-7′-dichloro-fluorescin diacetate (H_2_DCF-DA) is deacetylated by intracellular esterases to non-fluorescent H_2_DCF, which is trapped inside cells; when H_2_DCF is oxidized by H_2_O_2_ and other oxidant species, it turns into the highly fluorescent 2′-7′-dichloro-fluorescein (DCF). Briefly, cells were seeded in 12 or 24-well plates (each sample in triplicate) and treated as described in section 2.1. After 48 h of treatment, cells were quickly washed in ice-cold PBS and incubated for 45 min at 37 °C in phenol red-free RPMI 1640 containing 1 % FCS and 20 μM H_2_DCF-DA (Molecular probes, Eugene, OR). After cell washing in PBS and refilling the wells with fresh medium, fluorescence was measured in the microplate reader (Victor3, PerkinElmer) (excitation/emission wavelengths: 485/535 nm). ROS production was expressed as Arbitrary Units of Fluorescence (ΔAUF)/10^6^ cells. For each sample, a blank (fluorescence of cells without H_2_DCF-DA) was subtracted.

### 2.5 GSH determination

The intracellular level of GSH was measured essentially according to Sedlak and Lindsay [18]. Briefly, after cell harvesting by trypsinization, 10^6^ cells were washed in ice-cold PBS and carefully lysed in 150 μl of cold 5 % trichloroacetic acid (TCA). After centrifugation at 10,000*g* for 5 min at 4 °C, aliquots of acidic supernatant were assayed with the Ellman's Reagent, 5,5′-dithiobis (2-nitrobenzoic acid) (DTNB). GSH concentration was calculated as absorbance at 412 nm (on the basis of a proper GSH standard curve) and expressed as nmol/10^6^ cells.

### 2.6 Flow cytometry for macropinocytosis evaluation

Flow cytometry analysis was performed essentially as previously described [16]. Cells were seeded in 6-well plates (each sample in duplicate) and treated for 48 h as described in section 2.1; the medium used was phenol red-free RPMI, containing the fluid-phase tracer fluorescein isothiocyanate-conjugated Dextran 70 kDa (FITC-Dext) (46845, Merck) at the final concentration of 0.25 mg/ml. At the end of the experiment, FITC-Dext-containing medium was promptly removed, cells were washed three times with ice-cold PBS and harvested by trypsinization. Cells were pelleted by centrifugation (1,000*g* for 5 min), resuspended at the final concentration of 150 × 10^3^/ml in ice-cold PBS supplemented with 1 % FBS, and immediately analysed. A minimum of 10,000 cells per sample was analysed by flow cytometry using a Guava EasyCyte 6-2 L cytometer (Luminex Corporation), and data were analysed with FlowJo software (Tree Star). In each experiment, the fluorescence of proper blank samples (cells incubated without FITC-Dext) was also evaluated and subtracted from the experimental samples.

### 2.7 Clonogenic assay

The clonogenic assay was performed essentially according to a well-established procedure [19]. Three hundred fifty cells were seeded into 12-well plates (each sample in triplicate) and allowed to attach. The day after, cells were treated with different doses of trehalose (30, 60, 90 mM) and let to grow for 8–10 days in two different oxygen concentrations, as described in section 2.1. Cells were then washed with PBS and fixed for 10 min with paraformaldehyde (4 % in PBS); after two washing in PBS, cells were stained with crystal violet (0.5 % in 25 % methanol) and air-dried. For the colony counting, ImageJ software was set up to count clones containing at least 50 cells, as commonly accepted.

### 2.8 Phase contrast microscopy

Phase-contrast microscopy was used to evaluate cell morphology in the long term (8–10 days). Cells were seeded in 12-well plates (as done for the clonogenic assay) and treated as described in section 2.1. Images of live cells were captured on a Nikon Eclipse Ti microscope, equipped with DS-Q1Mc camera and a NIS element software (Nikon). For each sample, several randomly selected fields were photographed by using a 10X phase-contrast objective.

### 2.9 Statistics

Results are presented as mean with standard errors (±SE), and statistical significance was determined using one-way ANOVA followed by Tukey's multiple comparison test, or two-way ANOVA followed by Sidak's multiple comparison test. GraphPad Prism Software (Version 8.4.2) was used for statistical analyses. A *p* < 0.05 was considered as statistically significant.

## 3 Results

### 3.1 Activation of HIF-1α under hypoxia

**Fig. 1:**
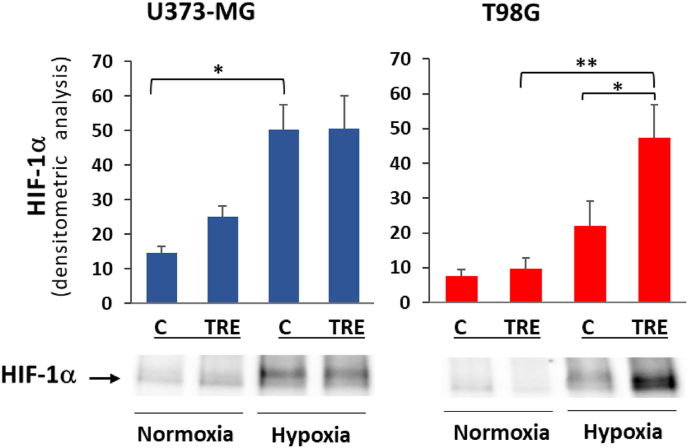
**Effect of hypoxia and trehalose treatment on HIF-1**α **levels.** U373-MG and T98G glioblastoma cells were untreated (C) or treated with 90 mM trehalose (TRE) for 48 h in normoxia or hypoxia. HIF-1α levels were measured in whole cell lysates by western blot. The intensity of protein band was normalized to the total amount of protein in each lane, as quantified using Image Lab software on Ponceau S-stained membrane. Results are presented as mean ± SE of 3 independent experiments. ∗*p* < 0.05, and ∗∗*p* < 0.01. Typical western blots are shown below the graphs.

The transcriptional factor HIF is the major hypoxia sensor and hypoxia response regulator. Under hypoxia, hydroxylation of the O_2_-sensitive HIF-α subunit by prolyl hydroxylases (PHDs) and the subsequent ubiquitin-dependent degradation of HIF-α are prevented, resulting in HIF-α accumulation and its heterodimerization with the constitutive HIF-1β subunit. Of the three isoforms of the HIF-α subunit (HIF-1α, -2α, and -3α), we monitored HIF-1α, as it is ubiquitously expressed in mammalian tissues. U373-MG and T98G glioblastoma cells, untreated or treated with trehalose 90 mM, were incubated for 48 h under normoxia and hypoxia, always in parallel. As shown in Fig. 1, cell hypoxia response was confirmed by accumulation of HIF-1α protein in all samples incubated under 1 % O_2_. In trehalose-treated T98G cells, such accumulation was even higher than in the corresponding untreated cells.

### 3.2 Expression of Nrf2 and HO-1 is lower under hypoxia

**Fig. 2:**
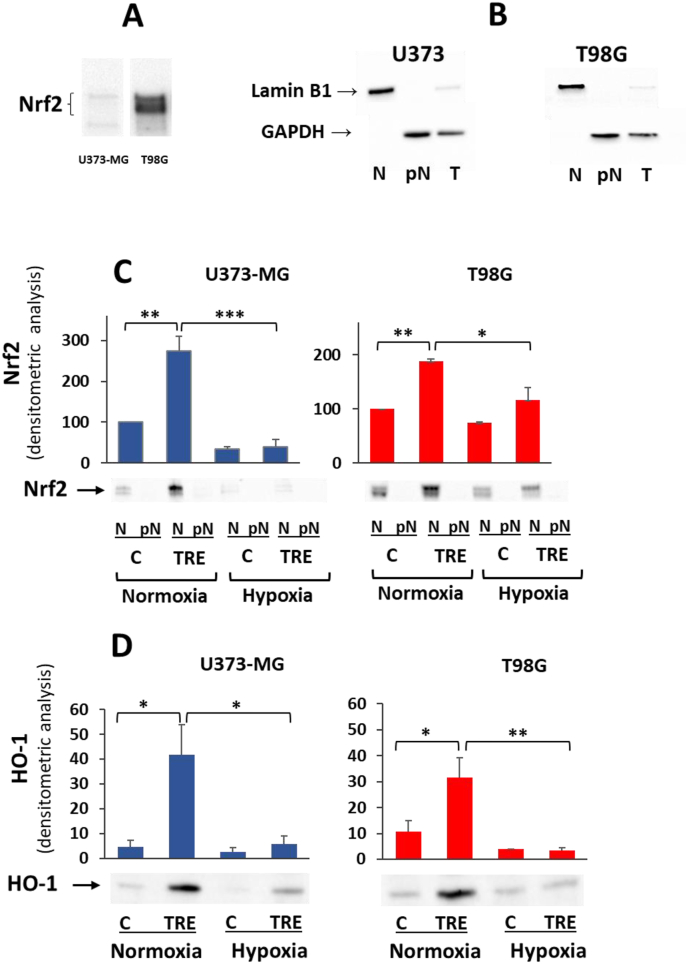
**The expression of Nrf2 and HO-1 is downregulated under hypoxia and upregulated by trehalose under normoxia. A.** Western blot of Nrf2 measured in whole cell lysates of control U373-MG and T98G cells cultured under normoxia. **B.** The nuclear marker Lamin B1 and the cytosolic marker GADPH were evaluated by western blot in nuclear fraction (N), post-nuclear fraction (pN), and whole cell lysate (T) of control U373-MG and T98G cells, to assess the quality of the subcellular fractionation procedure. **C.** U373-MG and T98G cells were untreated (C) or treated with 90 mM trehalose (TRE) for 48 h under normoxia or hypoxia, and the expression level of Nrf2 was evaluated by western blot in nuclear (N) and post-nuclear (pN) fractions. The intensity of protein band was normalized to the total amount of protein in each lane, as quantified using Image Lab software on Ponceau S-stained membrane. Results of nuclear Nrf2 levels are presented as mean ± SE of 3 independent experiments. ∗*p* < 0.05, ∗∗*p* < 0.01, and ∗∗∗*p* < 0.001. Typical western blots are shown below the graphs. **D.** In whole cell lysates of the above samples, the expression level of HO-1 was evaluated by western blot. The intensity of protein band was normalized to the total amount of protein in each lane, as quantified using Image Lab software on Ponceau S-stained membrane. Results are presented as mean ± SE of 3 independent experiments. ∗*p* < 0.05, and ∗∗*p* < 0.01. Typical western blots are shown below the graphs.

Since a positive relationship between transcription factors HIF-1α and Nrf2 is often reported in experimental models of cancer cells grown under hypoxia [20], we evaluated whether the hypoxia-dependent accumulation of HIF-α was parallelled by enhanced expression of Nrf2 in both cell lines. In preliminary experiments on control cells grown under normoxia, the basal level of Nrf2 protein evaluated in total cell lysates was very different in the two cell lines, being extremely low in U373-MG and very high in T98G cells (Fig. 2A), the latter cells harbouring amplification of the Nrf2-encoding gene, *NFE2L2* [21]. It is known that under basal conditions Nrf2 protein is sequestered in the cytoplasm, where its level is kept low by proteasomal breakdown. Under different stress conditions, cytosolic Nrf2 degradation is prevented, and this allows rapid translocation and accumulation of Nrf2 in nuclei.

In order to evaluate nuclear translocation of Nrf2, we preliminarily verified the quality of our subcellular fractionation protocol, in terms of enrichment of nuclear and post-nuclear fractions, in both cell lines. As expected, the nuclear membrane Lamin B1 protein was detected in total extract (T) (a faint band) and much more in the nuclear fraction (N), while the cytosolic enzyme GADPH was present in total extract and in the post-nuclear fraction (pN) (Fig. 2B).

Irrespective of the different basal levels in the two cell lines, Nrf2 was only present in nuclei and underwent similar changes under the same experimental settings in U373-MG and T98G cells (Fig. 2C). Under normoxia, treatment with trehalose significantly increased Nrf2 levels in both cell lines compared to untreated cells, the protein accumulating in the nuclear fraction. Under hypoxia, Nrf2 levels were significantly lower than under normoxia in U373-MG cells, whether untreated or trehalose-treated. Similarly, in hypoxic T98G cells, Nrf2 levels were lower than under normoxia; the decrease was less pronounced, presumably due to the very high basal levels of Nrf2 in these cells.

Since *HO-1* is a canonical Nrf2 target gene, HO-1 protein levels were also assessed to confirm Nrf2 activation (Fig. 2D). As expected, under normoxia, HO-1 protein increased significantly in both trehalose-treated cell types. In hypoxic U373-MG cells, the very low levels of HO-1 were also consistent with the low levels of nuclear Nrf2; likewise, in hypoxic T98G cells, HO-1 protein was very low, although Nrf2 levels only halved.

### 3.3 Cell redox imbalance occurs under hypoxia

**Fig. 3:**
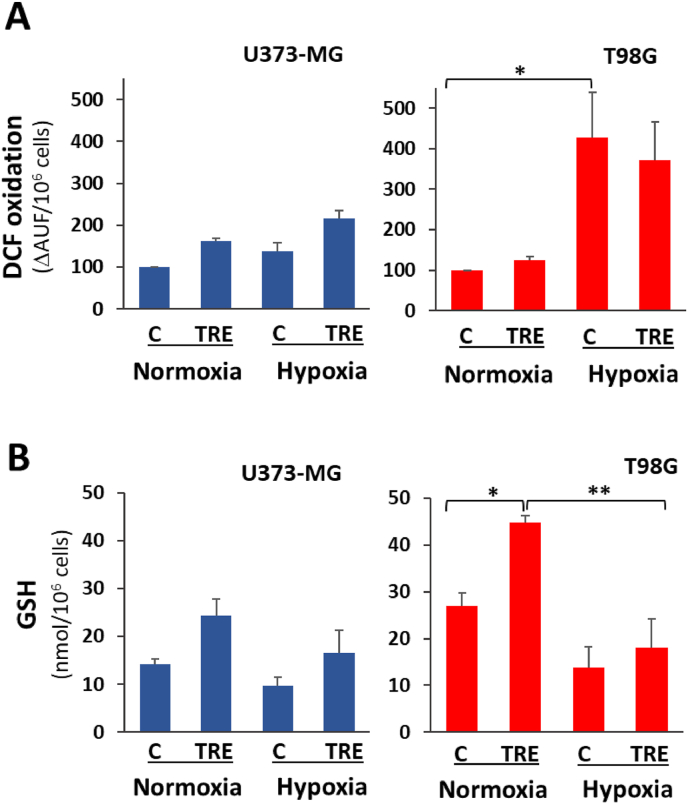
**Cellular redox state: ROS formation and GSH content are affected by hypoxia and trehalose treatment.** U373-MG and T98G cells were untreated (C) or treated with 90 mM trehalose (TRE) for 48 h under normoxia or hypoxia. **A.** Intracellular ROS formation was evaluated by DCF-based assay; DCF oxidation in control cells under normoxia is set to 100. Results are presented as mean ± SE of 3-5 independent experiments (each experiment performed on triplicate samples). **∗***p* < 0.05. **B.** Intracellular GSH content. Results are presented as mean ± SE of 4 independent experiments. ∗*p* < 0.05, and ∗∗*p* < 0.01.

Nrf2 is considered a major sensor of oxidative stress; once activated, it regulates cellular redox homeostasis through transcriptional induction of several enzymes acting as antioxidants at different levels. Thus, in order to directly evaluate cell redox status under normoxia and hypoxia, we measured intracellular formation of ROS in living cells by fluorometric DCF-based assay (Fig. 3A). The rate of ROS formation under basal conditions (i.e. no treatment and normoxia) was very different in the two cell lines, being 13.7 ± 3.7 and 6.3 ± 2.3 (mean ± SE) ΔAUF/10^6^ cells in U373-MG and T98G cells, respectively. In both cell lines, after 48 h of hypoxia, greater amounts of oxidant species were formed than under normoxia, the increase being remarkable both in untreated and trehalose-treated T98G cells. Moreover, in U373-MG cells, trehalose treatment alone stimulated a mild increase in ROS under normoxia and hypoxia.

We also measured the intracellular content of GSH (Fig. 3B), a major ubiquitous and abundant thiol antioxidant. GSH takes part in neutralization (by reduction) of ROS and peroxides through GSH-related enzymes, a few of which are transcriptionally induced by Nrf2. Firstly, in U373-MG cells GSH basal levels under normoxia were almost half that in T98G cells. Interestingly, this difference is consistent with the above-mentioned double content of ROS found in U373-MG cells with respect to T98G cells. In all samples incubated under hypoxia, GSH levels were lower than under normoxia; in particular, in T98G cells treated with trehalose, such a decrease is statistically significant. These results suggest that GSH may be consumed by the increased formation of ROS, as observed in all hypoxic samples and particularly in T98G cells. However, in trehalose-treated cells of both cell lines, under normoxia (and to lower extent under hypoxia), GSH levels were higher than in control cells. The increase in GSH is consistent with trehalose's established antioxidant role [22]; however, the mild increase of ROS levels observed in U373-MG cells remain unexplained at present.

### 3.4 Basal and trehalose-induced macropinocytosis are mildly affected by hypoxia

**Fig. 4:**
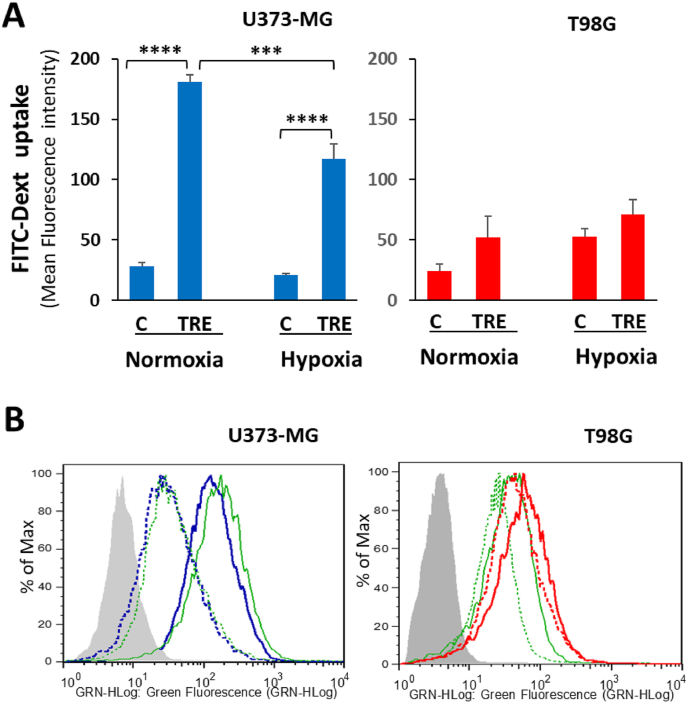
**Quantitative evaluation of macropinocytosis by flow cytometry.** U373-MG and T98G cells were untreated (C) or treated with 90 mM trehalose (TRE) for 48 h under normoxia or hypoxia; FITC-dextran 70 kDa (FITC-Dext) probe was present for the entire experimental time. **A.** Mean fluorescence intensity. Results are presented as mean ± SE of 3 independent experiments (each experiment performed on duplicate samples). ∗∗∗*p* < 0.001, and ∗∗∗∗*p* < 0.0001. **B.** Representative histogram plots of fluorescence. Grey areas: Blank sample (cells without probe); dashed lines: Control cells; solid lines: Trehalose-treated cells; green lines: Normoxia; blue lines (U373-MG cells) and red lines (T98G cells): Hypoxia.

Under standard normoxic cell culture conditions, we previously showed [16] that trehalose treatment induces remarkable dose-dependent macropinocytosis in U373-MG glioblastoma cells, whereas in T98G cells it does not induce macropinocytosis. Here, we asked whether hypoxia impacts macropinocytosis in both cell types. For this purpose, the uptake of the fluorescein-conjugated fluid-phase tracer, dextran 70 kDa (FITC-Dext), was measured by flow cytometry after 48 h of hypoxia or normoxia in untreated and trehalose-treated cells. As shown in Fig. 4, trehalose proved a strong inducer of macropinocytosis in U373-MG cells in normoxic conditions, and to a lesser extent under hypoxia. Conversely, dextran uptake by T98G cells under hypoxic conditions showed a slight increase in both control and trehalose-treated groups; however, in trehalose-treated cells, uptake remained significantly lower than that observed in U373-MG cells (*p* < 0.01, under both normoxic and hypoxic conditions).

### 3.5 Trehalose-induced autophagy is variably modulated by hypoxia

**Fig. 5:**
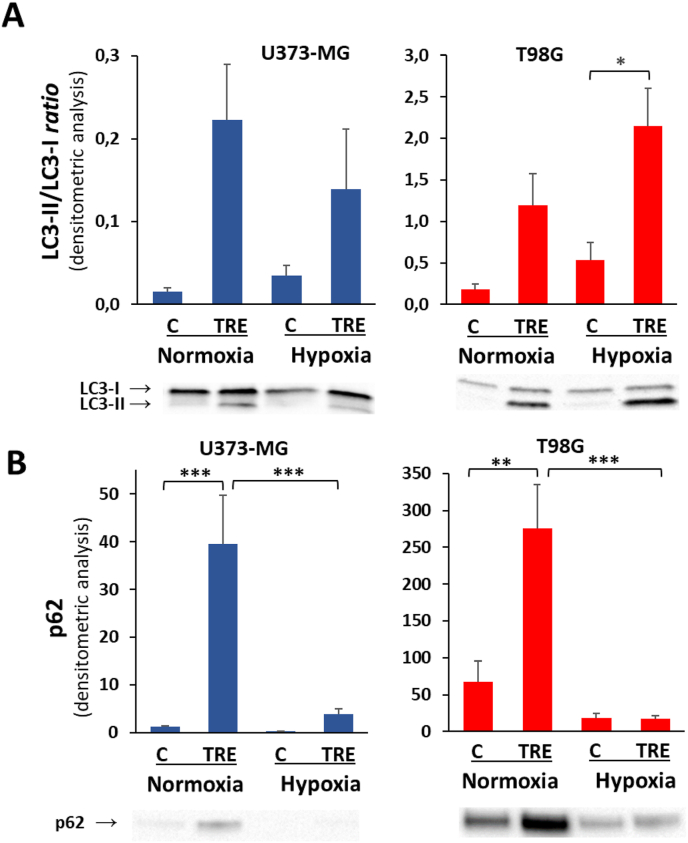
**The autophagy-related markers, LC3-II/LC3-I *ratio* and p62, are variably modulated by hypoxia.** U373-MG and T98G cells were untreated (C) or treated with 90 mM trehalose (TRE) for 48 h under normoxia or hypoxia. **A.** Autophagy was evaluated as *ratio* LC3-II/LC3-I measured in whole cell lysates by western blot. Results are presented as mean ± SE of 4 independent experiments. ∗*p* < 0.05. **B**. Western blot of SQSTM1/p62, measured in whole cell lysates. The intensity of protein band was normalized to the total amount of protein in each lane, as quantified using Image Lab software on Ponceau S-stained membrane. Results are presented as a mean ± SE of 5 independent experiments. ∗∗*p* < 0.01, and ∗∗∗*p* < 0.001. Typical western blots are shown below the graphs.

We previously demonstrated that trehalose-induced macropinocytosis and autophagy occurred in a mutually exclusive manner in U373-MG and T98G cells under normoxia [16]. Since hypoxia has been reported to enforce autophagic response in different experimental models of tumor cells [23], here we aimed to assess whether hypoxia also upregulated the autophagic process in glioblastoma cells, and whether the autophagic response under hypoxia was related to changes in macropinocytosis. Autophagy was evaluated as LC3-II/LC3-I *ratio*, the most widely used molecular marker of autophagy. During the autophagic process, LC3 native protein is first activated to LC3-I by Atg4-mediated proteolysis and then conjugated with phosphatidylethanolamine on autophagosome membranes, forming lipidated LC3-II; this binding remains throughout the pathway, from early autophagosome formation to its elongation and maturation. Thus, increased LC3-II levels and especially an increased LC3-II/LC3-I *ratio* reliably quantify the autophagic process. As shown in Fig. 5A, trehalose was confirmed to be a weaker autophagy inducer in U373-MG than in T98G cells, as shown by the ten-fold smaller y-axis scale recording LC3-II/LC3-I *ratio* in U373-MG cells; under hypoxic conditions, trehalose-induced autophagy was even lower. On the contrary, in T98G cells − proved to be autophagy-prone in normoxia − both basal and trehalose-induced autophagy was even higher under hypoxia than normoxia. As regards the relationship between macropinocytosis and autophagy, these processes underwent similar changes under hypoxic conditions: indeed, both decreased in U373-MG and increased in T98G cells.

To further assess the autophagic process, protein levels of p62/SQSTM1 (hereafter p62) are commonly assessed. p62 protein acts as a cargo receptor for autophagic degradation of polyubiquitinated target molecules, and in so doing, p62 itself is degraded in autophagolysosomes. Thus, decreased levels of p62 should reflect an ongoing autophagic process. However, in different experimental settings of autophagy stimulation, concomitant transcriptional induction of the *SQSTM1* gene occurs, as documented also by us in trehalose-treated melanoma cells [24]. It must therefore be considered that the steady-state level of p62 protein in trehalose-treated cells reflects both its autophagolysosome-dependent turnover and its neo-synthesis. As shown in Fig. 5B, in control normoxic cells there was a huge difference in the basal expression level of p62: in T98G cells it was approximately 50-fold that in U373-MG cells. Irrespective of this difference, trehalose treatment of both cell lines significantly increased p62 protein levels, presumably because its transcriptional induction outweighed its autophagy-mediated consumption. This effect was lost in trehalose-treated cells under hypoxia, where p62 fell dramatically.

### 3.6 LC3 and p62 proteins translocate in the cell nuclear fraction

**Fig. 6:**
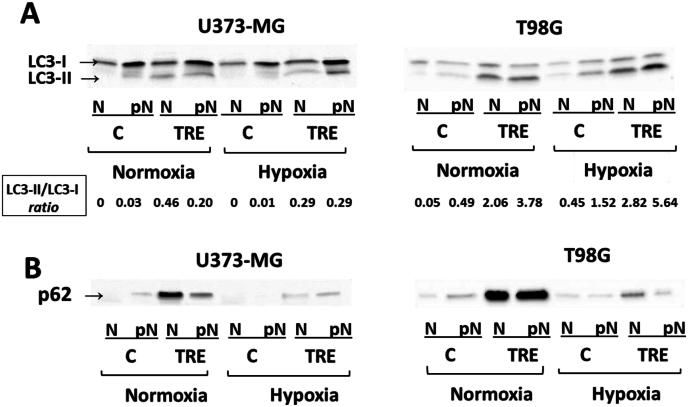
**A considerable amount of LC3 and p62 translocates in the nuclear fraction of trehalose-treated cells.** U373-MG and T98G cells were untreated (C) or treated with 90 mM trehalose (TRE) for 48 h under normoxia or hypoxia. **A.** Western blot of LC3 in nuclear (N) and post-nuclear (pN) fractions; LC3II/LC3I *ratio* was evaluated by densitometric analysis. **B.** Western blot of SQSTM1/p62 in nuclear (N) and post-nuclear (pN) fractions. Typical western blots are reported.

A particular finding was the presence of LC3 protein (LC3-I and LC3-II) and to a greater extent p62 protein in the nuclear fraction (N), along with their expected presence in the post-nuclear fraction (pN). As regards LC3 (Fig. 6A), we found an increased LC3-II/LC3-I *ratio* in the nuclear fraction of trehalose-treated U373-MG cells under normoxia, and a mild decrease of said ratio under hypoxia, as found in whole cell lysate (Fig. 5A). In the nuclear fraction of T98G cells as well, the LC3-II/LC3-I *ratio* mirrored that found in whole cell lysate (Fig. 5A): an increased LC3-II/LC3-I *ratio* in trehalose-treated cells in normoxic conditions, and a further increase of said *ratio* under hypoxia.

Similarly, p62 protein levels in the nuclear fraction (Fig. 6B) reflected those found in whole cell lysate (Fig. 5B) and in the post-nuclear fraction. In both cell lines treated with trehalose under normoxia, a huge amount of p62 was found in the nuclear fraction, even exceeding the post-nuclear fraction in U373-MG cells. Under hypoxia, where p62 levels in whole cell extract were very low in all samples (Fig. 5B), p62 still remained evident in nuclei of trehalose-treated cells.

### 3.7 Colony-forming capacity is affected by trehalose and hypoxia

**Fig. 7:**
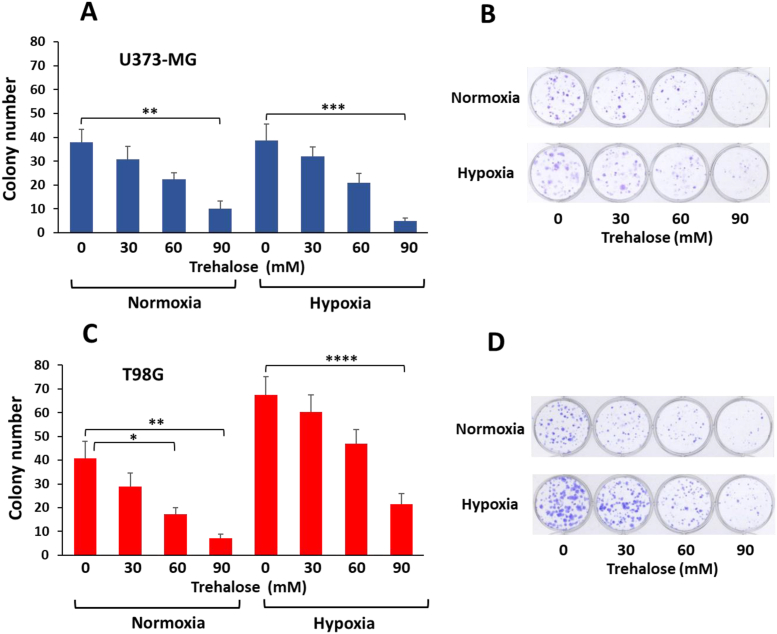
**Colony-forming capacity is affected by trehalose and hypoxia.** U373-MG and T98G cells were untreated (C) or treated with increasing concentrations (30, 60, and 90 mM) of trehalose (TRE) for 8-10 days under normoxia or hypoxia. **A.** and **C.** The clonogenic capability is expressed as mean number of colonies ± SE, from 3 (U373-MG cells) or 5 (T98G cells) independent experiments (each experiment performed on triplicate samples). ∗*p* < 0.05, ∗∗*p* < 0.01, ∗∗∗*p* < 0.001, and ∗∗∗∗*p* < 0.0001. Statistical significance was determined using two-way ANOVA followed by Sidak’s multiple comparison test. **B.** and **D.** Representative plates are shown for U373-MG and T98G cells, respectively.

In cells incubated for 8–10 days in hypoxic conditions, with or without trehalose, we evaluated reproductive cell survival by clonogenic assay, which indicates the capacity of very sparsely seeded cells to form clones. Compared to the little or no inhibition of cell proliferation after 48 h of trehalose or hypoxia treatment (data not shown), the cell response was remarkably different in the long term. In U373-MG cells (Fig. 7A and B), clonogenic efficiency was not affected by hypoxia, but was remarkably and dose-dependently impaired by trehalose treatment in a similar manner under both oxygen concentrations. The number of colonies in control T98G cells (Fig. 7C and D) under hypoxia was significantly higher than under normoxia. Trehalose treatment significantly and dose-dependently decreased clonogenic capacity, in roughly proportional manner under normoxia and hypoxia, though the absolute number of colonies in trehalose-treated cells under hypoxia remained notably higher than in those under normoxia.

### 3.8 Cell morphology after hypoxia and trehalose treatment in the long term

**Fig. 8:**
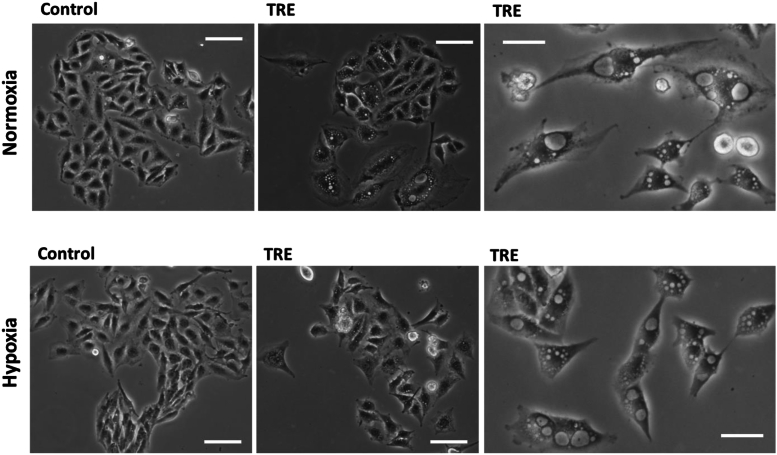
**In U373-MG cells the long-term treatment with trehalose induces cytoplasmic vacuolization and methuosis under normoxia and hypoxia.** U373-MG cells were untreated (Control) or treated with 90 mM trehalose (TRE) for 8-10 days under normoxia or hypoxia. Phase-contrast microscopy images of live cells were captured from plates set up as for the clonogenic assay. Left and central images: scale bar = 100 mm; right images: scale bar = 50 mm.

**Fig. 9:**
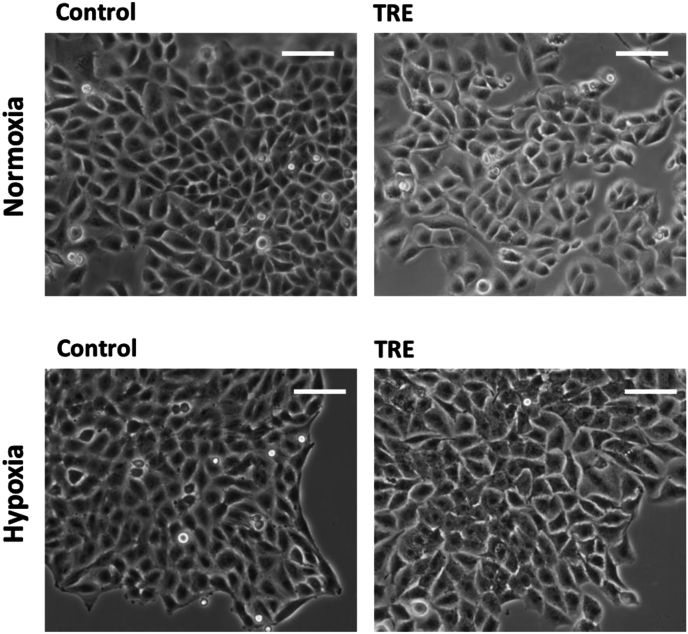
**T98G cells treated with trehalose and/or hypoxia in the long term.** T98G cells were untreated (Control) or treated with 90 mM trehalose (TRE) for 8-10 days under normoxia or hypoxia. Phase-contrast microscopy images of live cells were captured from plates set up as for the clonogenic assay. Scale bar = 100 mm.

In the long term (8–10 days), many U373-MG cells treated with trehalose (Fig. 8), particularly those isolated or in small colonies, showed the morphological changes typical of extensive macropinocytosis, including swelling, flattened peripheral regions, and refringent vacuoles of various sizes occupying most of the cytoplasm. Other cells contained very large vacuoles (presumably derived from end-stage coalescence of smaller vesicles), or were rounded and detached, indicating cells dying or killed by methuosis, respectively (Fig. 8, right panels). Although dextran uptake was lower in trehalose-treated cells under hypoxia than normoxia at shorter experimental times (48 h), the above morphological pictures were only slightly less frequent and intense, but overall very similar. In T98G cells (Fig. 9), the typical morphological features of macropinocytosis, not to mention cell death by methuosis, were minor or absent, and trehalose-treated cells generally appeared healthy at both oxygen concentrations. Hypoxia alone did not affect the cell morphology of either cell line.

## 4 Discussion

In scientific laboratories all over the world, cells are cultured under controlled air conditions considered standard, i.e. in a humidified atmosphere with 5 % CO_2_. This condition corresponds to an oxygen concentration of approximately 18 % and is regarded as normoxia. However, the mean oxygen level in living mammalian tissues is estimated at around 5 %; even in the airways, lung epithelium has O_2_ levels of about 14 %, and the lumen of intestinal mucosa is practically anaerobic. A low oxygen content is traditionally referred to as “hypoxia”, this term implying a pathological state. Due to the naturally occurring low oxygen content of most tissues, this point of view has been challenged by various scientists, and the recently introduced term “physioxia” is indeed more suitable for indicating “physiological hypoxia” [2]. The supraphysiological oxygen levels typically used in standard cell culture could therefore actually be a “hyperoxic” stimulus, which might compromise the effectiveness of *in vitro* models in reproducing *in vivo* conditions. The question is even more relevant in *in vitro* models of tumor cells, since due to uncontrolled cell proliferation and/or insufficient vasculature, oxygen levels in most solid tumors are much lower than in normal tissues, ranging from 0.5 % to 2 % [[3], [4], [5]], and such “physiological hypoxia” is a chronic condition. With the exception of central areas of tumor tissue that often undergo necrosis due to prolonged deep hypoxia (<0.01 % O_2_), tumor cells adapt well to hypoxia and survive. Hypoxia is even thought to drive malignant progression, and chemo-, radio- and immune-resistance [3,25]. As in most solid tumors, O_2_ levels are lower (below 2 %) in glial tumors [4,5] than in healthy brain tissue (around 5 %) [26]. In these tumors the hypoxia-driven advantage – in terms of malignant progression and resistance to therapy – is considered relevant [27,28].

In the present study, we evaluated the effect of hypoxia on two human glioblastoma cell lines, U373-MG and T98G, in the presence of trehalose, previously demonstrated to stimulate different biological responses under normoxia [16]. As expected, HIF-1α was activated in all cells incubated under hypoxia. The regulation of HIFs depends on the degradation or stabilization of the oxygen-dependent subunits HIF-1/2/3α under normoxia or hypoxia, respectively. Under high oxygen tension, the major mechanism of HIF-1α down-regulation consists in hydroxylation of specific proline residues by oxygen-sensitive prolyl hydroxylases (PHDs), followed by recognition of HIF-1α by von Hippel Lindau protein (pVHL), and subsequent ubiquitination and proteasomal degradation. Under low oxygen tension, mainly due to inhibition of PHDs, the fraction of ubiquitinated HIF-1α decreases dramatically, resulting in accumulation of the protein, which dimerizes with the constitutively expressed subunit HIF-1β [20]. PHDs are also sensitive to cell redox state; in fact, among other mechanisms, increased levels of ROS can oxidize cysteine residues in the catalytic domain of PHD2, which significantly decreases its hydroxylation activity on HIFs-α [29].

The glioblastoma microenvironment is characterized by hypoxia and high levels of ROS [30]; consistently, in both the glioblastoma cell lines used, hypoxia induced an increase in ROS production in untreated and trehalose-treated cells. Although controversial [31], mitochondria are considered to be the major source of ROS, which are generally derived from superoxide radical anions (${O_{2}}^{-}$) [32]. In particular, when molecular oxygen is low, two-electron reduction of O_2_ to H_2_O is impaired, and the one-electron reduction carried out by respiratory complex III produces ${O_{2}}^{-}$; mitochondrial superoxide dismutase (SOD2) subsequently catalyses dismutation of ${O_{2}}^{-}$ to H_2_O_2_, which ultimately exits mitochondria. In our experimental model, the increased levels of ROS occurring in hypoxia can be expected to play a role in HIF-1 activation. Interestingly, we find that HIF-1α stabilization in hypoxic T98G cells was remarkably higher in trehalose-treated than control cells, despite similar ROS levels. This gain in HIF-1 activation opens up two interpretations: a) as documented in a few cancer metabolism studies [33,34], HIF signalling can also be induced by various oxygen-independent mechanisms, collectively termed “pseudohypoxia”; thus, in trehalose-treated T98G cells, hypoxia-dependent ROS formation and pseudohypoxic mechanisms could act jointly; b) some negative regulators of HIF1-α could be depleted by the sustained autophagic process (as discussed later). Here, the increased ROS levels produced in both cell lines during hypoxia were coupled with a lower intracellular content of the reduced form of glutathione (GSH). Since GSH is a major cellular antioxidant in charge of neutralizing ROS and ROS-derived peroxides by reduction, GSH consumption is presumably related to its scavenging activity towards ROS. These combined events (particularly evident in T98G cells) therefore confirm occurrence of a cell redox imbalance under hypoxia. Later, on the basis of the cell fate observed, we discuss whether this redox imbalance can be considered physiological oxidative stress (eustress) or a harmful event (oxidative distress).

In trehalose-treated cells under normoxic conditions, and to a lesser extent under hypoxia, GSH levels were higher than in untreated cells. This observation is consistent with trehalose's antioxidant role, as documented in various experimental models [22], and is primarily attributed to activation of the non-canonical SQSTM1/p62–Keap1–Nrf2 signaling axis: trehalose upregulates *SQSTM1* expression in terms of mRNA and p62 protein [24,35,36], presumably through Transcription Factor EB (TFEB) [37], known to transactivate several genes involved in lysosome biogenesis and autophagy. p62 interacts with Keap1 (which is ultimately degraded inside autophagolysosomes), thus competing for binding of Keap1 with Nrf2 in the cytosol [38]. Since Keap1 negatively regulates Nrf2, the competitive interaction of p62 with Keap1 elicits Nrf2 stabilization and nuclear translocation; Nrf2 binds with antioxidant responsive elements (ARE) of ARE-containing genes, such as genes coding enzymes involved in GSH synthesis (*GCLC*, *GCLM*, and *SLC7A11*) and GSH recycling from GSSG (*GSR1*) [39]. Interestingly, Nrf2 also induces expression of its own positive regulator, *SQSTM1*/p62, thereby fuelling a positive feedback loop in the SQSTM1/p62-Keap1-Nrf2 pathway [40].

Here, we demonstrated occurrence of the above molecular relationship in trehalose-treated cells. Indeed, Nrf2 accumulates in nuclei under normoxic conditions, and p62 is also increased proportionally with respect to untreated cells; conversely, the depletion of p62 occurring in all hypoxic samples, along with decreased activation of Nrf2, may indicate the loss of the positive feedback loop between Nrf2 and *SQSTM1*/p62. Interestingly, a remarkable moiety of p62 accumulated in nuclei, at levels that exactly reflect those found in whole cell lysate. At present, we have no explanation for this translocation within the current experimental framework. Given that the interaction of p62 with Keap1 and Nrf2 primarily occurs in the cytosol [40], we cannot exclude the possibility that the enhanced nucleocytoplasmic shuttling of p62 induced by trehalose treatment may be involved in other mechanisms, either autophagy-dependent or -independent, including ubiquitin/proteasome-mediated protein turnover within the nucleus [41].

The canonical activation of Nrf2, considered a major sensor of oxidative stress, depends on oxidation of critical cysteine residues in its redox-sensitive regulator, Keap1; the resulting conformational change in Keap1 protein allows Nrf2 to escape from sequestration in the cytosol and proteasomal degradation through cullin-3-dependent ubiquitination [42]. On this basis, Nrf2 was expected to be activated under hypoxia, where cells experience a condition of redox imbalance; on the contrary, not only did we find no activation of Nrf2, but Nrf2 activation was lower than under normoxia, albeit to a different extent. This lack of Nrf2 activation during hypoxia recalls the already mentioned “non-canonical” SQSTM1/p62-Keap1-Nrf2 axis [38]. In line with this, a significant positive correlation between *SQSTM1* mRNA/p62 protein and Nrf2 activity has been observed in a panel of glioblastoma cell lines and in samples of glioblastoma, particularly of the more aggressive mesenchymal subtype [21]. As expected from the lowering of Nrf2 in hypoxia, a positive correlation between Nrf2 and HIF-1 was also lacking in control and trehalose-treated cells. The complex molecular events triggered by hypoxia (in the tumour milieu as well as *in vitro*) are responsible for an intricate interplay between HIF and Nrf2. Indeed, a positive or inverse correlation between HIF and Nrf2 activation that depends on the cell and experimental context has been documented [20].

Given the many molecular changes occurring in glioblastoma cells under hypoxia (with or without trehalose), the question is how these molecular changes impact two important cellular events, i.e. macropinocytosis and autophagy. There is very little in the literature on the role of hypoxia in the macropinocytotic response of tumor cells; to the best of our knowledge, only two studies have reported stimulation of macropinocytosis by hypoxia in cancer cells (pancreatic and hepatic) [43,44]. In our hands, a minor macropinocytosis occurred in glioblastoma T98G cells under hypoxia. On the contrary, in glioblastoma U373-MG cells, trehalose treatment under normoxia induced striking macropinocytosis, which was lower, albeit still remarkable, under hypoxia. The autophagic response under hypoxia was also non-canonical: the trehalose-induced autophagy under hypoxia in U373-MG cells was even lower than under normoxia, confirming that these cells are also autophagy-deficient under conditions of HIF-1 upregulation, reported to enforce the autophagic response [45,46]. Conversely, in autophagy-proficient T98G cells, the effect of hypoxia was conventional; in fact autophagy increased in control cells and even more so in trehalose-treated cells. Irrespective of the different autophagic responses in the two cell lines, there was a peculiar significant amount of LC3 in the nuclear fraction of trehalose-treated cells, where the *ratio* LC3-II/LC3-I strictly reflected that found in whole cell lysate and in the post-nuclear fraction. Although various functions of nuclear shuttling of LC3 have been proposed in different experimental models [47], we currently have no mechanistic or functional explanation for this event in the present cellular context.

The chronic hypoxic condition that glioblastoma cells experience in the brain is generally considered to favour cell growth and malignant progression. In our experimental model of hypoxia, the questions are: whether cells adapt to or are harmed by redox imbalance, macropinocytosis and autophagy; and what the fate of glioblastoma cells is in the long term. More colonies were only formed under hypoxia in control T98G cells, suggesting that these cells benefit from oxygen shortage. Based on its high expression of Nrf2 and p62, this cell line is considered to resemble the more aggressive mesenchymal subtype of glioblastoma [21]; the same authors also demonstrated that inhibition of Nrf2 and p62 by siRNAs impaired cell proliferation. On the contrary, we found that hypoxia-induced down-regulation of Nrf2 and p62 accompanied increased colony-forming capacity. This apparent discrepancy confirms that the hypoxic response is cell-context-specific, and that hypoxia-driven molecular changes must be evaluated as a whole. In this regard, we believe that T98G cells under hypoxia experience oxidative eustress [48], namely finely tuned physiological production of ROS, which act as signalling agents and can benefit tumor cells in various ways, including cell proliferation. Particular emphasis has been placed on the role of hypoxia in the generation and self-renewal of glioblastoma stem-like cells (GSCs) [[49], [50], [51]]. Accordingly, we hypothesize that hypoxia-driven oxidative eustress benefits the population of GSCs presumably present in the T98G cell line, by improving their capacity to form clones.

The canonical mTOR-dependent autophagic process stimulated under hypoxia (in basal conditions or drug-induced) is considered a survival mechanism, also involved in intrinsic or acquired therapy resistance [52]. Indeed, autophagy is a double-edged sword, and much evidence demonstrates that by stimulating excessive self-digestion beyond the limits of cell viability, exogenously hyperactivated autophagy can even promote cell demise in various cancer types, including glioblastoma [8,[53], [54], [55]]. Consistently, in T98G cells treated with trehalose (an mTOR-independent autophagy inducer), reduced clonogenic capacity rules out any protective role of activation of autophagy under normoxia and even more so under hypoxia, although the latter condition turns out to be beneficial *per se* for untreated cells. Since trehalose-treated T98G cells generally appear healthy, we believe that (over)stimulation of autophagy exerts a cytostatic effect in this context [56], rather than inducing autophagy-dependent cell death (ADCD) or autophagy-mediated cell death (AMCD). Unlike for T98G cells, hypoxia and the respective cell redox imbalance do not appear to be favorable conditions for U373-MG cells. In these autophagy-refractory cells, we believe that extensive and prolonged activation of macropinocytosis triggered by trehalose in the long term causes cell death by methuosis, whether under normoxia or hypoxia. To the best of our knowledge, macropinocytosis has never been studied in glioblastoma cells under hypoxia; indeed, (hyper)stimulation of macropinocytosis is virtually doubly tumor suppressive by facilitating entry of anticancer drugs into cells [57,58], and/or by inducing cell death by methuosis.

## 5 Conclusions

We believe that experimental models of glioblastoma cells grown under low oxygen concentrations are appropriate and necessary, as hypoxic *in vitro* conditions reproduce the tumor microenvironment much better than normoxic conditions. Also, such cell models could give an albeit partial picture of the variable and still unsatisfactory response of glioblastomas to current postoperative standard-of-care treatment; in fact, U373-MG and T98G human glioblastoma cells are relatively sensitive and resistant to temozolomide [59] and ionizing radiation [60], respectively. In our hands, hypoxia only appeared to be advantageous for T98G cells, however trehalose treatment impaired the clonogenic potential of these as well as U373-MG cells. The ability of trehalose to stimulate sustained autophagy or macropinocytosis may play a tumor-suppressive effect under physiological hypoxic conditions. Furthermore, future studies will aim to investigate whether trehalose can also act synergistically with conventional chemotherapy and radiotherapy, as we previously demonstrated in human melanoma cells [24]. To date, several trials assessing the safety and therapeutic potential of systemic administration of trehalose can be found in the Clinical Trials database (https://clinicaltrials.gov/) [15]. Since many local drug delivery strategies that directly release drugs into the brain have been promisingly investigated for the treatment of glioblastoma [[61], [62], [63]], it would be worth exploring the potential of trehalose as a therapeutic compound for local administration.

There are two limitations in this study that could be addressed in future research. First, a mechanistic validation would be needed to better analyze the role of trehalose-induced autophagy or macropinocytosis; for this purpose, suitable siRNAs and/or pharmacological inhibitors of these processes are needed, which do not show nonspecific cytotoxicity and exert a long-lasting inhibitory effect. Second, a preclinical mouse model of glioblastoma would be useful to confirm the antitumor efficacy of trehalose *in vivo*, through systemic or local administration.

## CRediT authorship contribution statement

**Barbara Del Bello:** Visualization, Validation, Methodology, Investigation, Formal analysis, Data curation, Conceptualization. **Cristina Ulivieri:** Methodology, Investigation, Formal analysis. **Emilia Maellaro:** Writing – review & editing, Writing – original draft, Validation, Supervision, Project administration, Investigation, Funding acquisition, Formal analysis, Data curation, Conceptualization.

## Funding

This work received financial support (to E. Maellaro) by PSR (Piano di Sostegno alla Ricerca 2023–2024) of University of Siena. The sponsor was not involved in the study design, in the collection, analysis and interpretation of data, in the writing of the report, and in the decision to submit the article for publication. APCs were partially covered by University of Siena, which is gratefully acknowledged.

## Declaration of competing interest

I have nothing to declare.

## Data Availability

Data will be made available on request.

## Glossary

HIF-1α: Hypoxia-Inducible Factor-1α
Nrf2: Nuclear factor erythroid 2-Related Factor 2
GADPH: Glyceraldehyde-3-phosphate dehydrogenase
HO-1: Heme-Oxygenase 1
ROS: Reactive Oxygen Species
GSH: reduced Glutathione
LC3: Microtubule-associated protein 1A/1B-light chain 3
p62/SQSTM1: Sequestosome-1
Atg4: Autophagy-related protein 4
PHDs: Prolyl hydroxylases
pVHL: von Hippel-Lindau protein
SOD2: mitochondrial superoxide dismutase
FITC-Dext: fluorescein-conjugated dextran 70 kDa
