# Supplementary material for: Hypoxia response in glioblastoma cells: effect of trehalose on macropinocytosis, autophagy and cell survival
Source: Biochem Biophys Rep. 2025 Oct 2;44:102284. doi: 10.1016/j.bbrep.2025.102284 (PMC12524557; doi:10.1016/j.bbrep.2025.102284)
Supplement: Multimedia component 1 [file mmc1.pdf]

- For Western blot images, ChemiDoc™ XRS+ apparatus was set to merge the chemiluminescence signal from protein bands with the colorimetric signal from pre-stained molecular weight standards.
- In some images the range of molecular weight standards is not complete; this means that, just after electrophoretical transfer, the nitrocellulose membrane was cut into two pieces in order to reveal different proteins in the same gel.
- Samples not inserted in red rectangles were analysed for different purposes and not included in this study.
- Blots referred to as Replicate 1 have been shown as typical blots in the figures.

Fig. 1 - HIF-1α

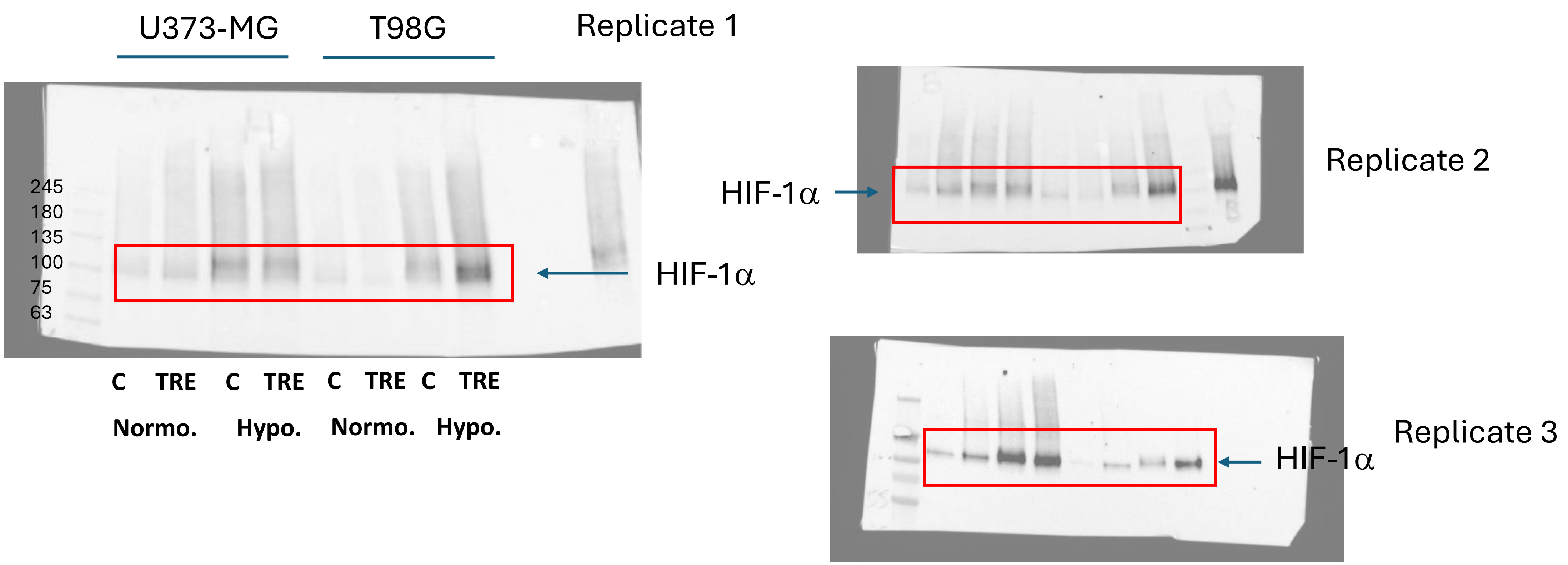

Fig. 2 B - Lamin B and GADPH

U373-MG

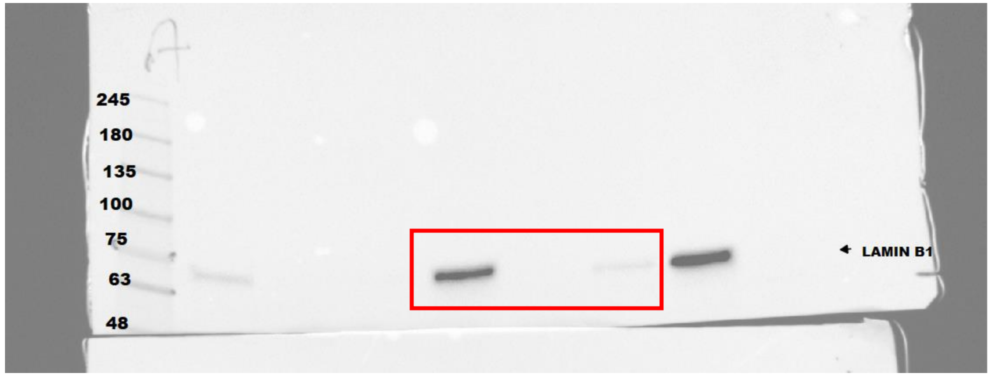

N pN T

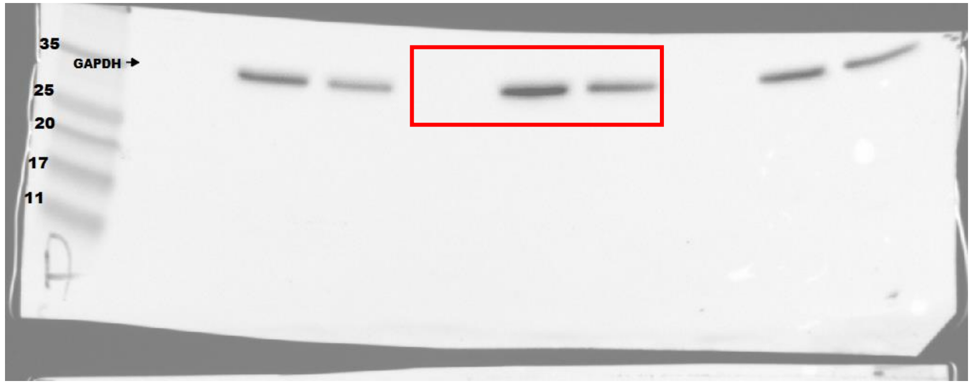

T98G

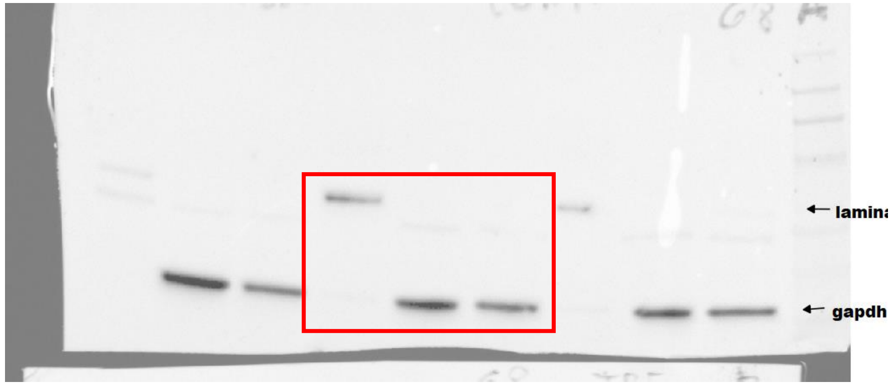

N pN T

Fig. 2 C - NRF2

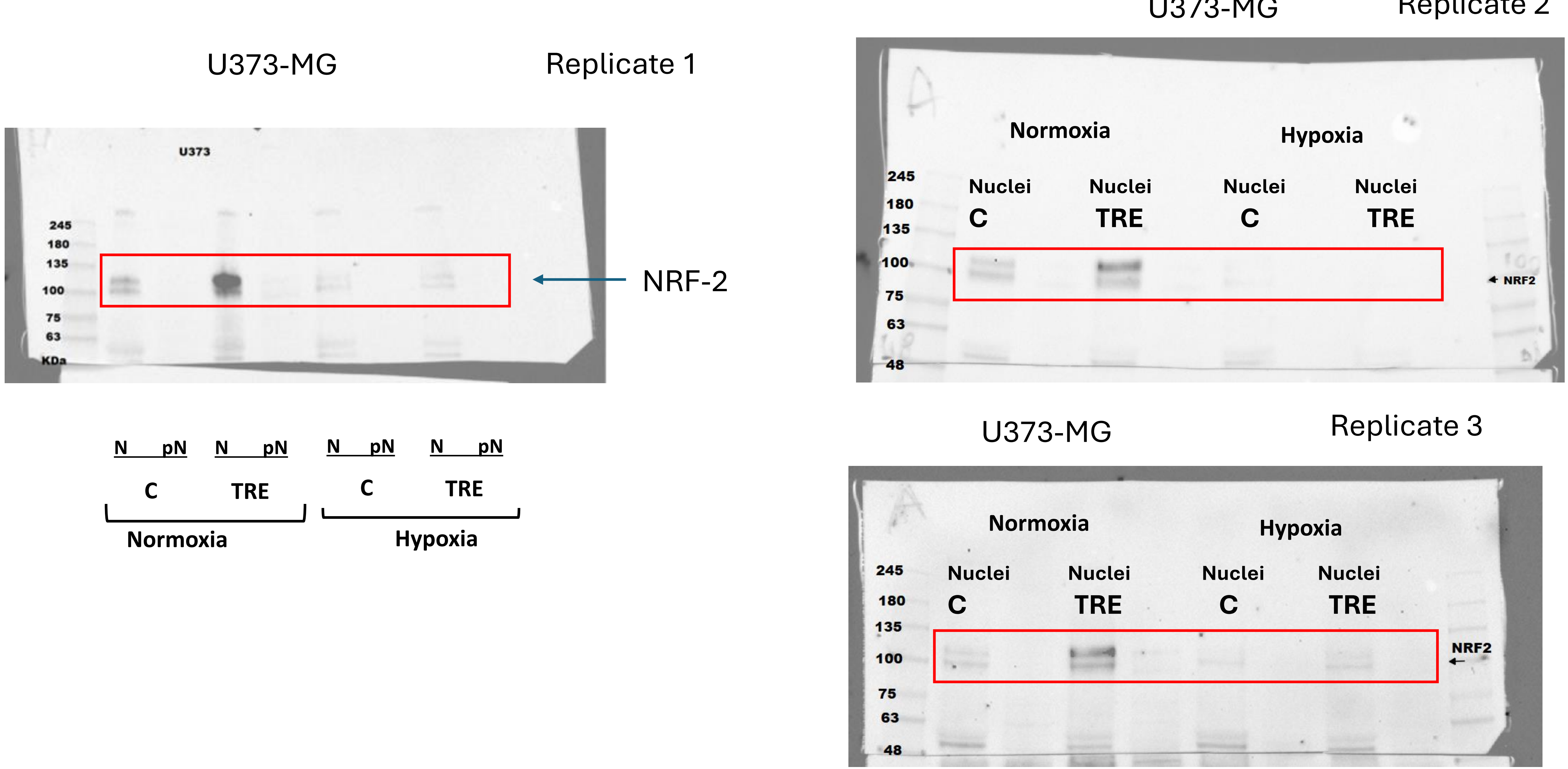
U373-MG Replicate 1
U373
245
180
135
100
75
63
KDa
NRF-2
N pN N pN N pN N pN
C TRE C TRE
Normoxia
Hypoxia
U373-MG Replicate 2
Normoxia
Hypoxia
Nuclei C
Nuclei TRE
Nuclei C
Nuclei TRE
245
180
135
100
75
63
48
NRF2
U373-MG Replicate 3
Normoxia
Hypoxia
Nuclei C
Nuclei TRE
Nuclei C
Nuclei TRE
245
180
135
100
75
63
48
NRF2

Fig. 2 C - NRF2

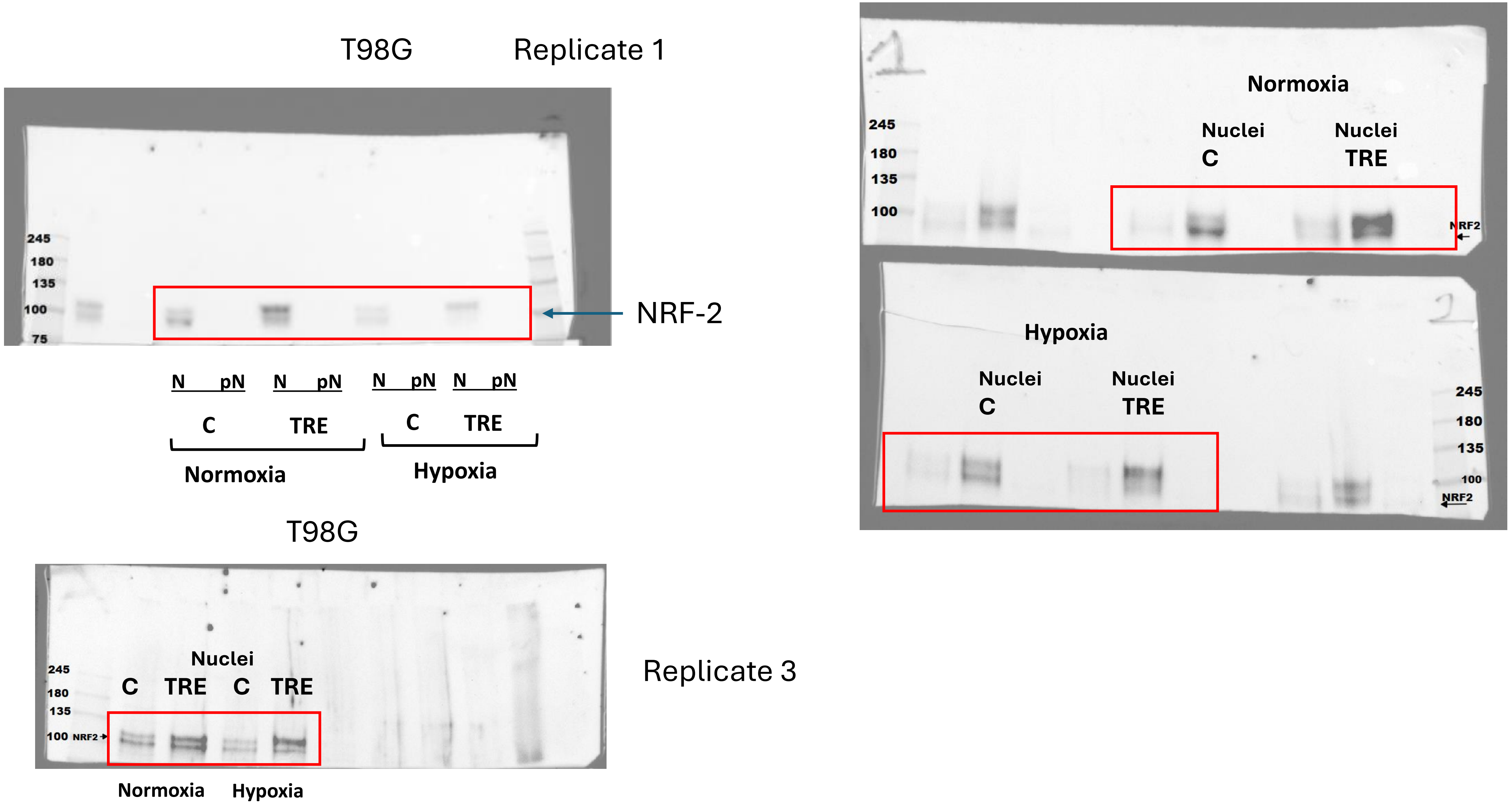

T98G
Replicate 2
T98G
Replicate 1
245
180
135
100
75
NRF-2
N pN N pN N pN N pN
C TRE C TRE
Normoxia
Hypoxia
Normoxia
Nuclei
C
Nuclei
TRE
245
180
135
100
NRF2
Hypoxia
Nuclei
C
Nuclei
TRE
245
180
135
100
NRF2
T98G
245
180
135
100 NRF2
Nuclei
C TRE C TRE
Replicate 3
Normoxia
Hypoxia

Fig. 2D - HO-1

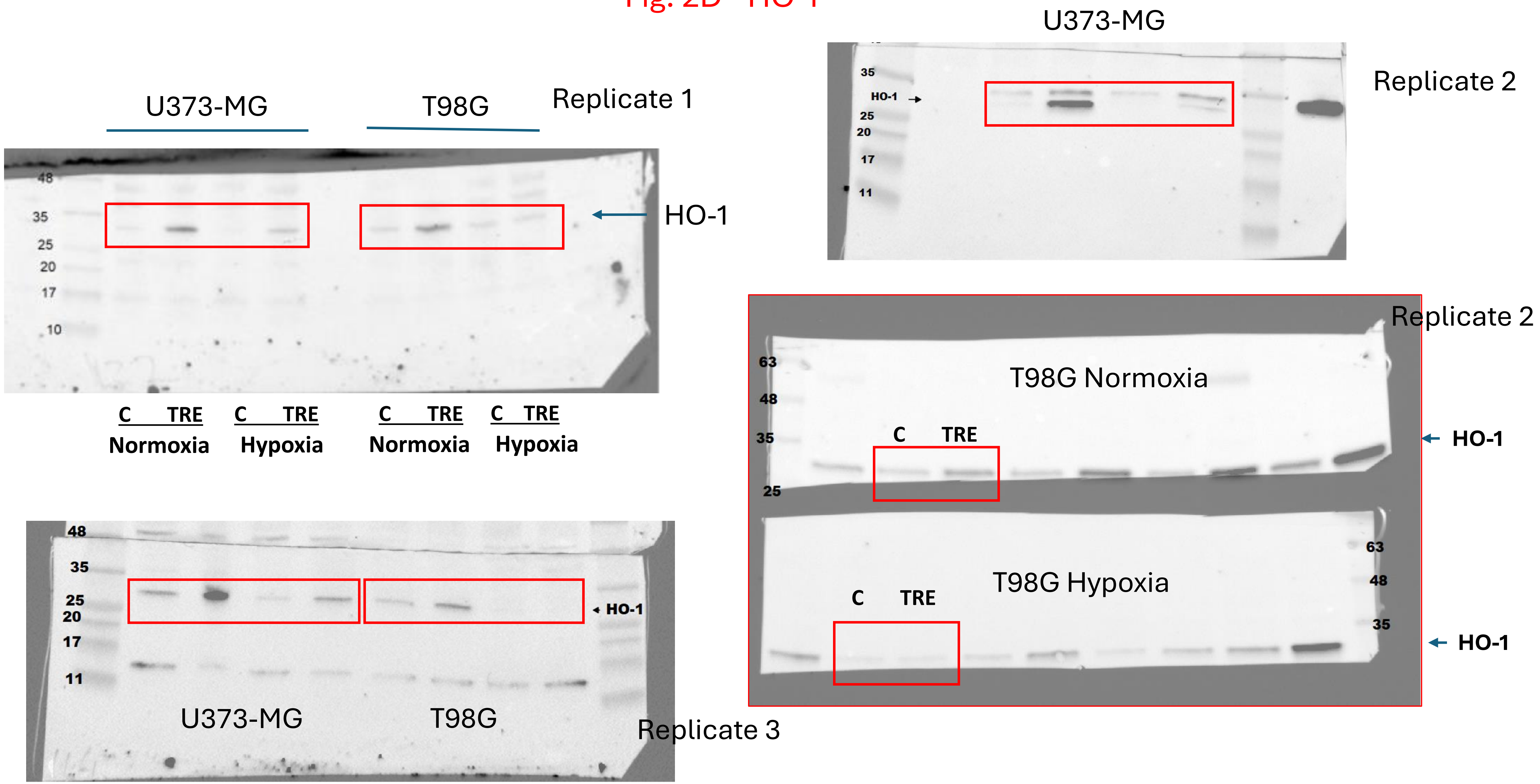
U373-MG
T98G
Replicate 1
48
35
25
20
17
10
HO-1
C TRE
Normoxia
C TRE
Hypoxia
C TRE
Normoxia
C TRE
Hypoxia
48
35
25
20
17
11
HO-1
U373-MG
T98G
Replicate 3
U373-MG
35
HO-1
25
20
17
11
Replicate 2
Replicate 2
63
48
35
25
T98G Normoxia
C TRE
HO-1
63
48
35
T98G Hypoxia
C TRE
HO-1

Fig. 5 A – LC3

U373-MG

Replicate 1

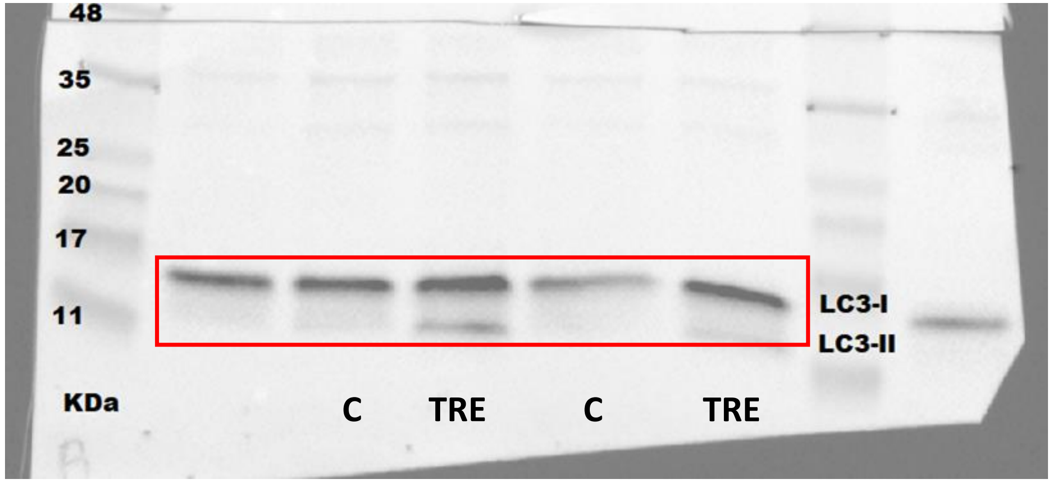

Normoxia Hypoxia

T98G

Replicate 1

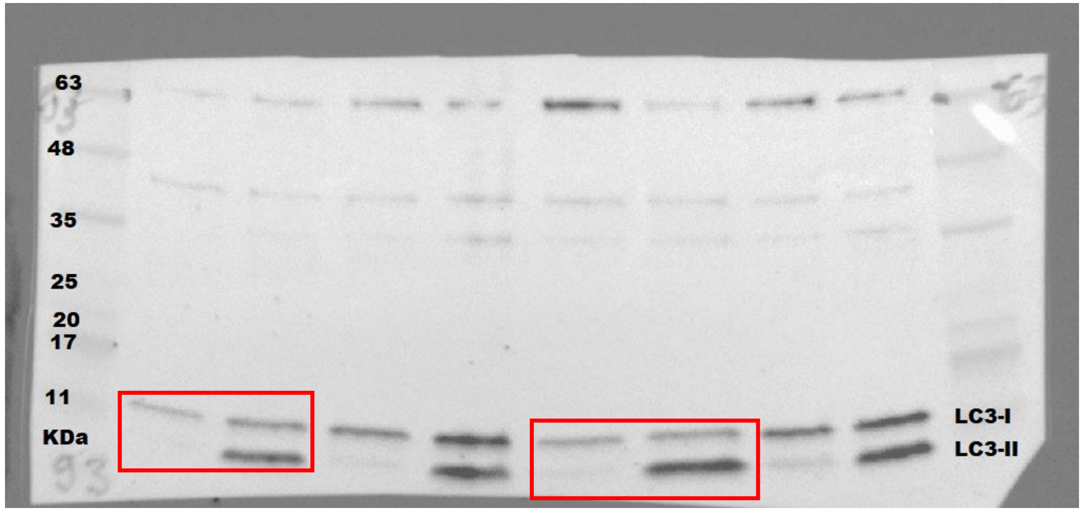

C TRE

Normoxia

C TRE

Hypoxia

Fig. 5 A – LC3

Replicate 2

U373-MG

Replicate 2

T98G

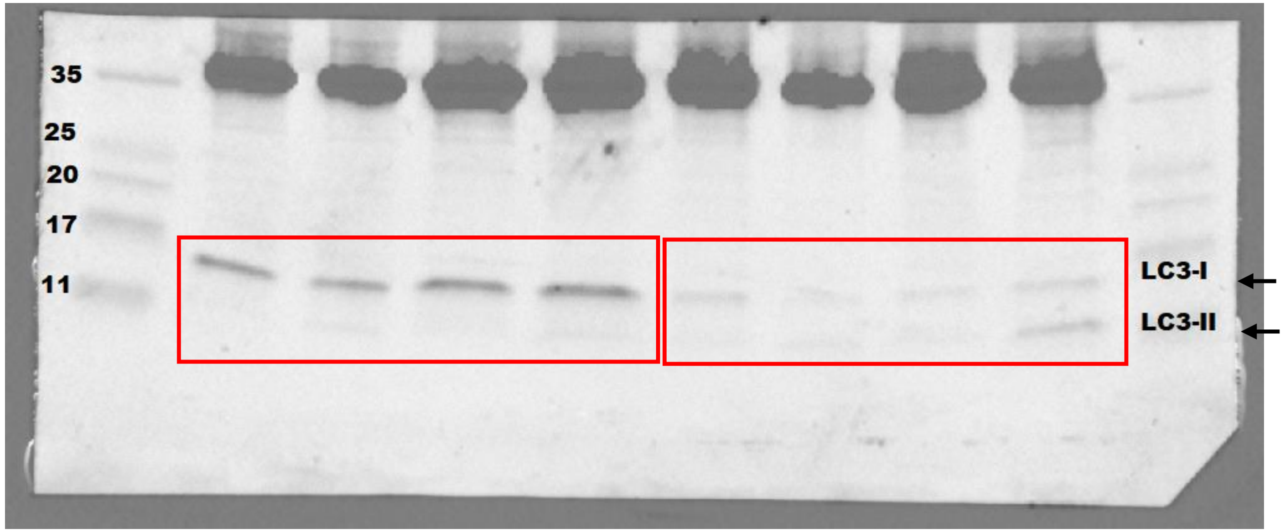

Replicate 3

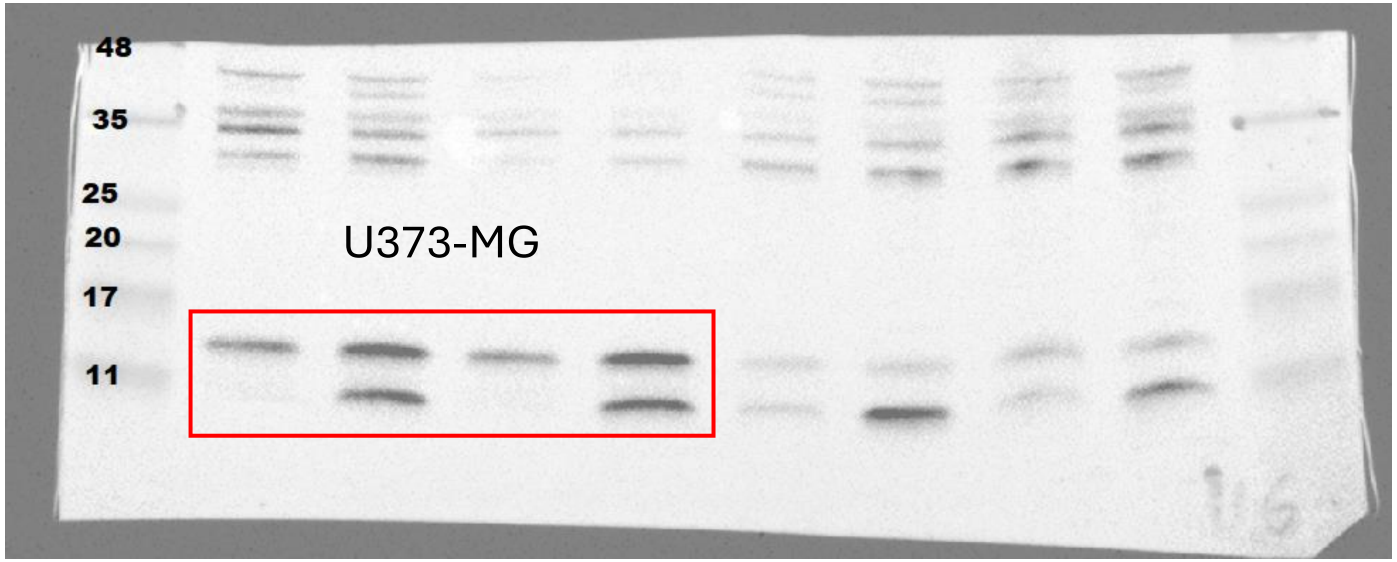

T98G Replicate 3

Fig. 5 A – LC3

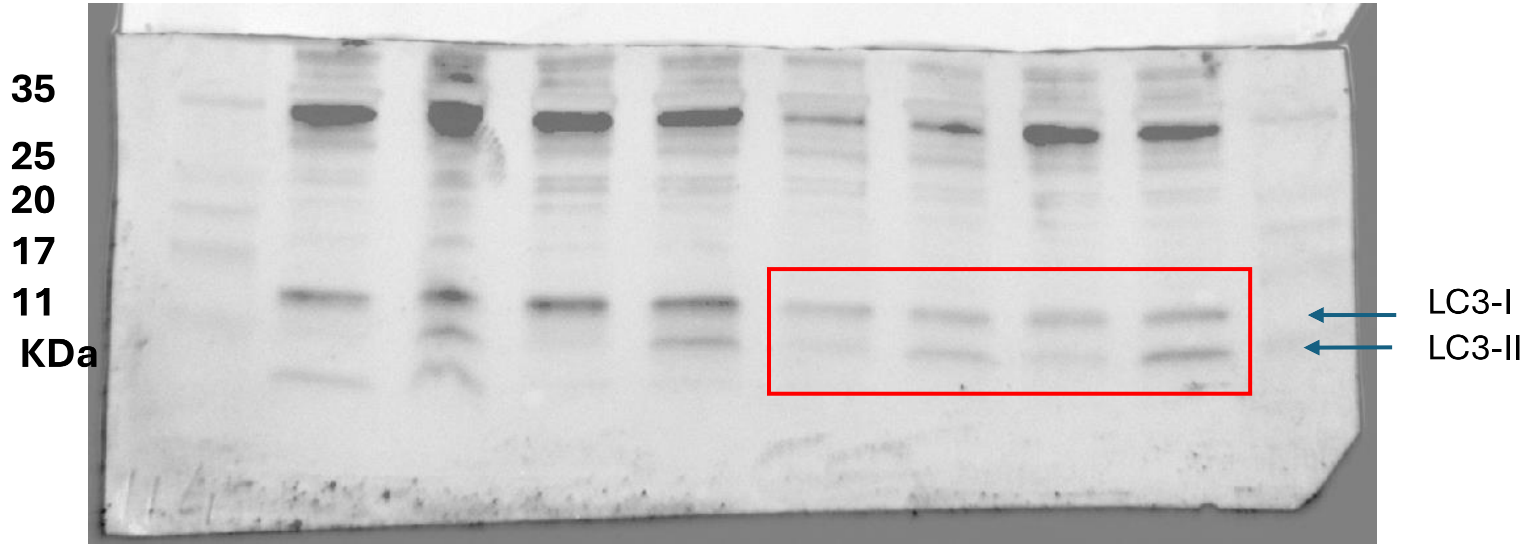

T98G Replicate 4

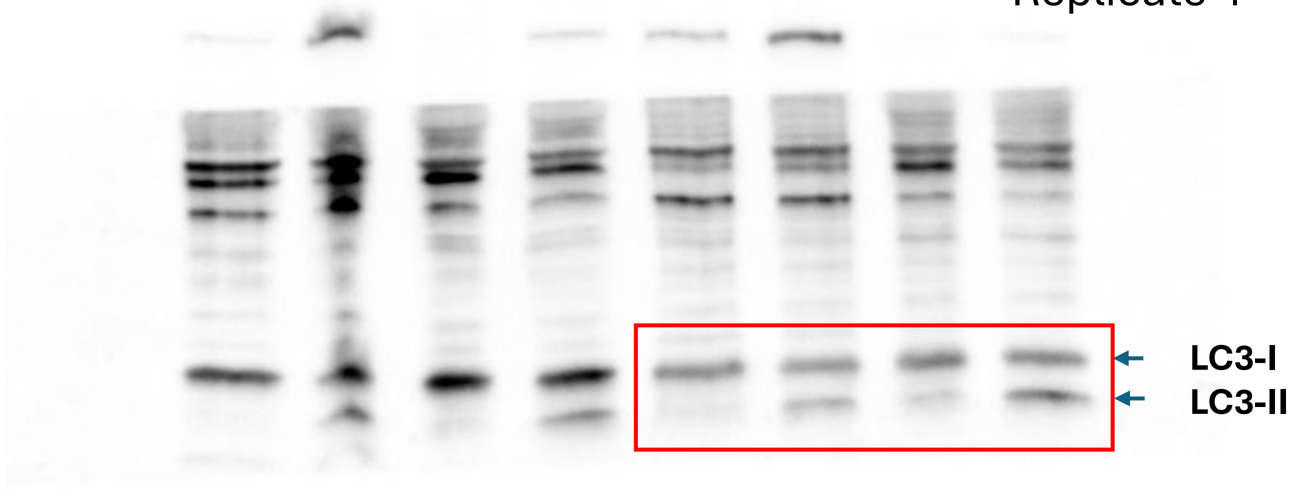

Fig. 5 B - p62

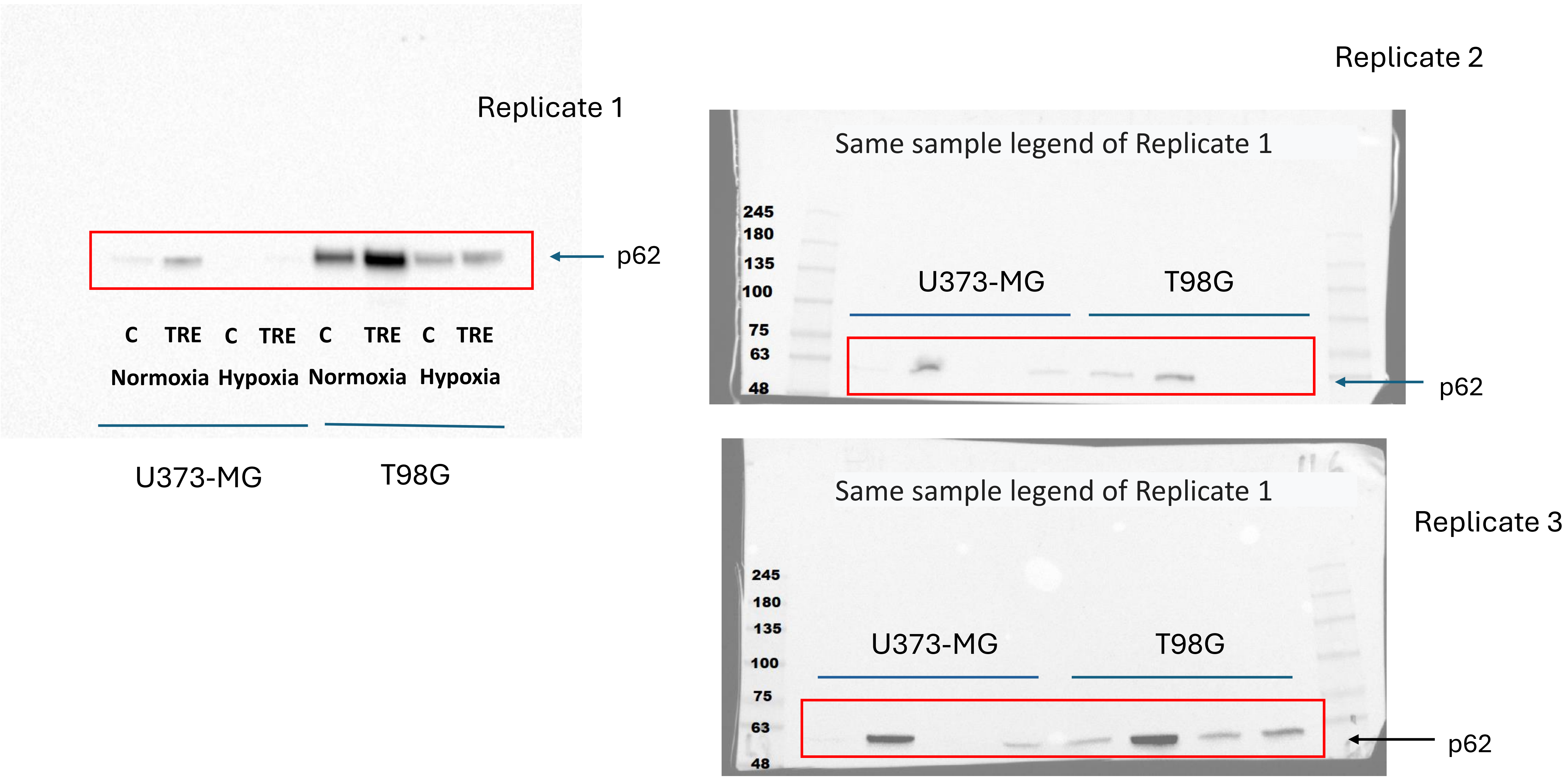

Replicate 1
p62
C TRE C TRE C TRE C TRE
Normoxia Hypoxia Normoxia Hypoxia
U373-MG
T98G
Replicate 2
Same sample legend of Replicate 1
245
180
135
100
75
63
48
U373-MG
T98G
p62
Same sample legend of Replicate 1
Replicate 3
245
180
135
100
75
63
48
U373-MG
T98G
p62

Fig. 5 B - p62

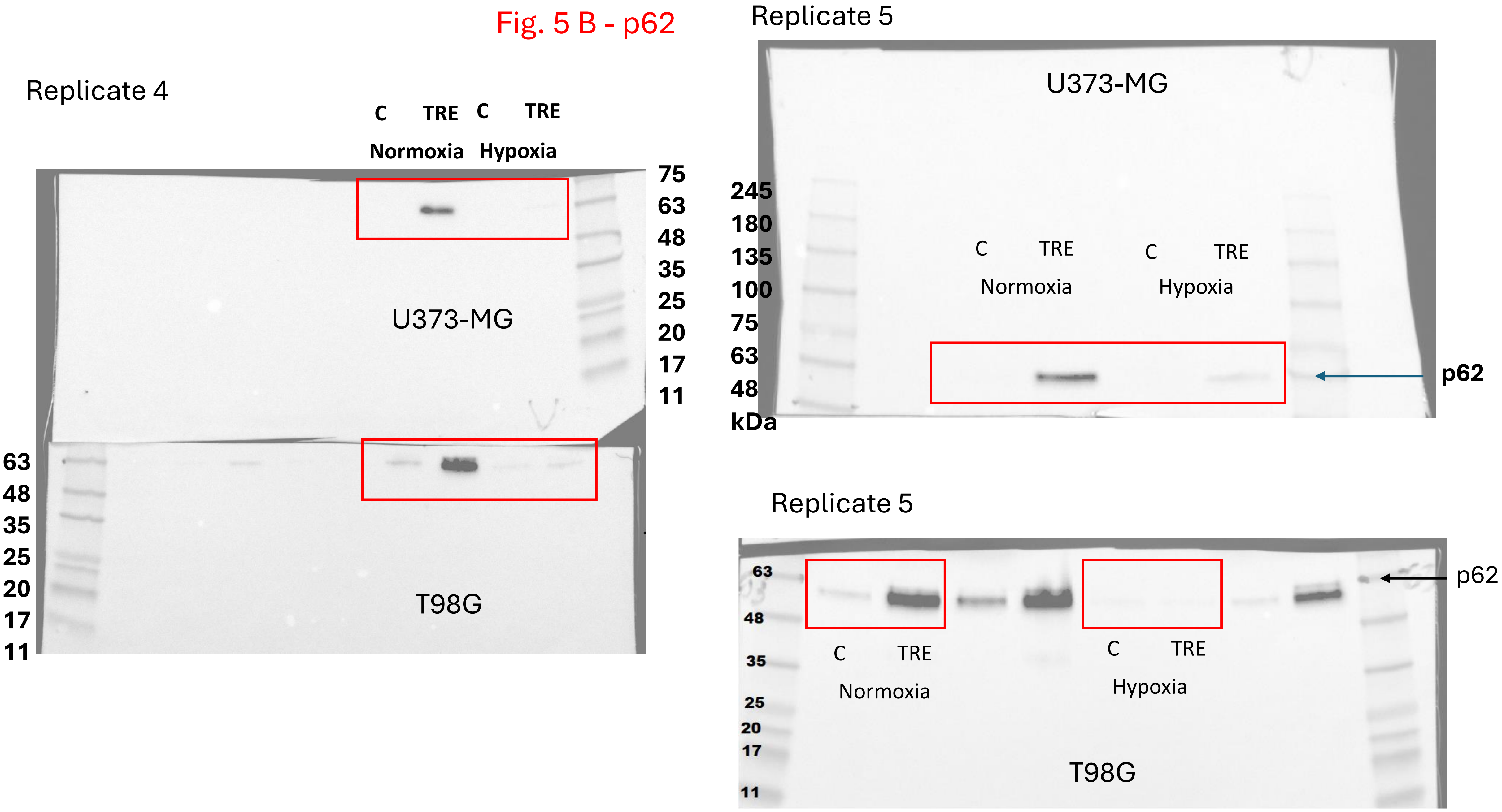
Replicate 4
C
TRE
C
TRE
Normoxia
Hypoxia
U373-MG
75
63
48
35
25
20
17
11
T98G
63
48
35
25
20
17
11
Replicate 5
U373-MG
245
180
135
100
75
63
48
kDa
C
TRE
C
TRE
Normoxia
Hypoxia
p62
Replicate 5
63
48
35
25
20
17
11
C
TRE
Normoxia
C
TRE
Hypoxia
T98G
p62

Fig. 6

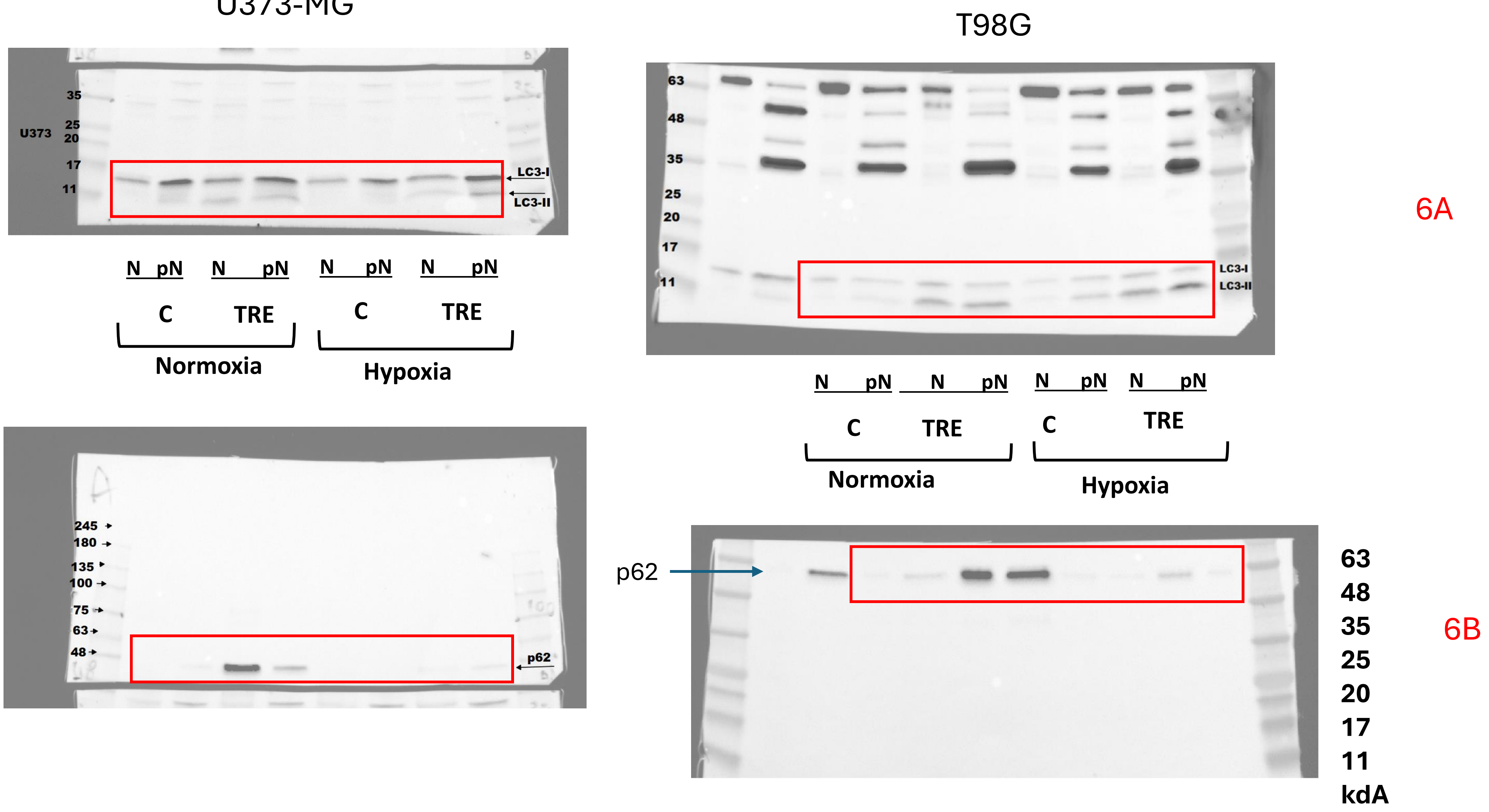

U373-MG
T98G
35
25
20
17
11
U373
LC3-I
LC3-II
N pN N pN N pN N pN
C TRE C TRE
Normoxia
Hypoxia
63
48
35
25
20
17
11
6A
245
180
135
100
75
63
48
p62
6B
63
48
35
25
20
17
11
kdA
